# Goodness of fit tests for random multigraph models

**DOI:** 10.1080/02664763.2022.2099816

**Published:** 2022-07-21

**Authors:** Termeh Shafie

**Affiliations:** GESIS – Leibniz Institute for the Social Sciences, Cologne, Germany

**Keywords:** Network model, multivariate networks, data aggregation, random multigraphs, goodness of fit, random stub matching

## Abstract

Goodness of fit tests for two probabilistic multigraph models are presented. The first model is random stub matching given fixed degrees (RSM) so that edge assignments to vertex pair sites are dependent, and the second is independent edge assignments (IEA) according to a common probability distribution. Tests are performed using goodness of fit measures between the edge multiplicity sequence of an observed multigraph, and the expected one according to a simple or composite hypothesis. Test statistics of Pearson type and of likelihood ratio type are used, and the expected values of the Pearson statistic under the different models are derived. Test performances based on simulations indicate that even for small number of edges, the null distributions of both statistics are well approximated by their asymptotic $\chi^{2}$-distribution. The non-null distributions of the test statistics can be well approximated by proposed adjusted $\chi^{2}$-distributions used for power approximations. The influence of RSM on both test statistics is substantial for small number of edges and implies a shift of their distributions towards smaller values compared to what holds true for the null distributions under IEA. Two applications on social networks are included to illustrate how the tests can guide in the analysis of social structure.

## 1. Introduction

In the domain of statistical network modeling, formal principles of hypothesis testing to assess goodness of fit are scarce. Most existing methods are computational and depend on visual inspections [9,14,21,26], with the exception of advances made for stochastic block models where principled statistical inference are developed by Lei [17], and those contingent on large sample properties (see, e.g. [2,20]). In this paper, we present and study the performance of goodness of fit tests for some probabilistic models on small undirected multigraphs with applications on social networks.

A multigraph is defined as a graph where multiple edges and self-edges (edge loops) are permitted [22]. Such data structures are either directly observed in settings where several edges can be mapped on the same vertex pair, or obtained by different forms of data aggregation. Directly observed data structures represented as multigraphs are also referred to as multi-relational, multiplex or multilayered networks [4,8,13,16,18]. Examples include social networks with multiple types of relations (e.g. friendship, collaborations, advice sharing) or the same relation with varying intensity (e.g. frequency of social exchange or interaction). These multigraph representations commonly exclude self-edges.

When the data includes actor or vertex covariates, multigraphs can be obtained by aggregating vertices into special subsets based on classified or cross-classified actor attributes. These types of aggregations are reviewed and exemplified in [7,22,23]. The reduced aggregated multigraphs may be of much smaller size than the initial graph since blocks of vertices are aggregated into single meta nodes. Further, within block edges are here represented as edge loops. These small multigraphs are the focus of this paper. This is due to the computational complexity of simulating larger sized multigraphs, but also due to a renewed interest in the statistical analyses of structure and compositions of small scale social networks arising from, e.g. families, personal networks, work teams and other small social groups [27].

A random multigraph model is given by a probability distribution over some class of multigraphs. In this article, two multigraph models introduced in [22,23] are considered. The first model is obtained by random stub matching with fixed degrees (RSM) so that edge assignments to vertex pair sites are dependent, and the second is obtained by independent edge assignments (IEA) according to a common probability distribution. Further, we present two different methods for obtaining an approximate IEA model from an RSM model. This is done by assuming that the stubs are randomly generated and independently assigned to vertices (ISA) and can be viewed as a Bayesian model for the stub frequencies under RSM. Another way of obtaining an approximate IEA model is to ignore the dependency between edges in the RSM model and assume independent edge assignments of stubs (IEAS). As shown in [23], the analysis of local and global properties of multigraphs is significantly simplified if the IEA approximations can be used. Thus, it is of particular interest to statistically analyze and test differences and similarities between these two models.

In order to assess goodness of fit, we use measures between the edge multiplicity sequence of an observed multigraph under RSM or IEA, and the expected multiplicity sequence according to a simple or composite IEA hypothesis. Test statistics of Pearson type and of information divergence type are used, and the expected values of the Pearson goodness of fit statistic under the different multigraph models are derived. The exact distributions of the test statistics are numerically investigated and compared to different approximations given by adjusted $\chi^{2}$-distributions which are useful for power analysis. Several test illustrations are included, both for tests of simple and composite hypotheses. The results from these test illustrations are used to guide the applicability of the tests to real world data.

Some problems we want to specifically analyze are how the test statistics behave for small number of edges and compare their behavior under RSM and IEA. This will provide insight into their applicability in real world settings and whether the test results are reliable. Critical regions of the goodness of fit statistics with a given significance level according to their asymptotic distributions are chosen, and answers to questions like the following are searched for: are the actual significance levels of the test statistics for small number of edges far from significance level of the asymptotic distribution? Is the convergence of the cumulative distribution functions of the test statistics slow or rapid? Does it depend on specific parameters in the models? Can better approximations to the actual distributions be obtained by using information about moments and adjustments of the $\chi^{2}$-distributions? Can power approximations be made for test statistics for small number of edges? How is power related to parameters of the models? How can RSM be tested and how does RSM influence the distributions of the goodness of fit statistics?

This paper as organized as follows. In Section 2, some basic notations are introduced and the different multigraph models mentioned above are defined, including the two ways in which an approximate IEA model can be obtained from an RSM model. Statistical tests of simple hypotheses are considered in Section 3, where the hypotheses are fully specified IEA models. For an IEAS model, the edge probability parameters are functions of a specified degree sequence, and for an ISA model these parameters are functions of a specified stub selection probability sequence. The Pearson goodness of fit statistic *S* and the divergence statistic *A* for these tests are defined and the expected value of *S* is derived under the different multigraph models. In particular it is shown that for the null distribution under RSM, this expected value only depends on the numbers of vertices and edges. In Section 4, tests of composite multigraph hypotheses are considered for IEAS, ISA and RSM models. The composite multigraph hypotheses are unspecified IEAS or ISA where the parameters have to be estimated from data. Test illustrations based on simulations for IEAS, ISA and RSM models are presented in Section 5 and supplementary material, where powers, moments and cumulative distribution functions of the test statistics are used to compare and evaluate their performances. All tests are performed using the R package multigraphr [24]. In Section 6, we summarize and discuss the general results from the simulated tests, and compare the performances of the two test statistics with respect to their error probabilities. Following this section, two real world applications are given in Section 7 to highlight the potential and versatility of the tests. We conclude with a general discussion regarding the results and possible extensions to the presented study, where suggestions on how the tests can be extended to also include RSM hypotheses and on how the tests can be made applicable for larger multigraphs.

## 2. Random multigraph models

We start by introducing some notations. A finite graph *g* with *n* labeled vertices and *m* labeled edges associates with each edge an ordered or unordered vertex pair. Let V={1,…,n} and E={1,…,m} be the sets of vertices and edges labeled by integers, and let *R* denote the set of available sites for the edges. For directed graphs the site space is $R=V^{2}$ and the number of sites is given by $r=n^{2}$. For undirected graphs we use the site space $R=\{(i,j)\in V^{2}:i\leq j\}$ where we consider (i,j) with i≤j as a canonical representation for the unordered vertex pair. The number of sites for undirected graphs is given by r=(n+12). The graph is thus an injective map $g:E\to R\subseteq V^{2}$.

A random multigraph is given by a probability distribution over some class of multigraphs. A multigraph with labeled vertices and undistinguished edges is represented by the random edge multiplicity sequence $M=(M_{ij}:(i,j)\in R)$ where the edge multiplicity $M_{ij}$ denotes the number of multiple edges at site (i,j)∈R. For undirected multigraphs, the edge sites are listed in the canonical order (1,1)<(1,2)<⋯<(1,n)<(2,2)<(2,3)<⋯<(n,n), so that $M_{ii}$ is the number of loops at vertex *i*, and $M_{ij}$ for *i*<*j* is the number of edges between vertices *i* and *j*. In this case it is convenient to define $M_{ij}=0$ for *i*>*j*. The edge multiplicity sequence M has total

(1) $$M_{\cdot \cdot }=\underset{i\leq j}{\sum \sum }M_{ij}=m$$

and

(2) $$M_{i\cdot }+M_{\cdot i}=\sum_{j=1}^{n}M_{ij}+\sum_{j=1}^{n}M_{ji}=d_{i}$$

is the degree of vertex *i*, which can also be interpreted as the number of edge-stubs or half-edges at vertex *i* for i=1,…,n. The stub multiplicity sequence $d=(d_{1},\ldots ,d_{n})$ has total $\sum_{i=1}^{n}d_{i}=2m$.

Consider a random undirected multigraph model where the edges are independently assigned to sites according to a common probability model [22]. Let $Q_{ij}$ denote the probability of assigning an edge to site (i,j)∈R so that ${\sum \sum }_{i\leq j}Q_{ij}=1$. This independent edge assignment (IEA) model has edge multiplicity sequence M(IEA) that is multinomially distributed with parameters *m* and $Q=(Q_{ij}:(i,j)\in R)$ so that the observed edge sequences M=m have probabilities [22]

(3) P(M(IEA)=m)=(mm)Qm=m!∏i≤jmij!∏i≤jQijmij.

Another random multigraph model is obtained by assuming that the edges are formed by random matching of pairs of edge stubs (half edges) in a given sequence of stubs [23]. This random stub matching (RSM) model has fixed stub multiplicity sequence $d=(d_{1},\ldots ,d_{n})$. Under RSM, the edge assignments to sites are dependent. The probability that an edge is assigned to site (i,j)∈R is given by

(4) Qij={(di2)/(2m2)for i=jdidj/(2m2)for i<j,

so that the edge probability sequence Q=Q(d) is a function of the stub multiplicity sequence d. The probability of edge multiplicity sequence m under RSM is shown in [23] to be given by

(5) P(M(RSM)=m)=2m2(mm)(2md)=2m2m!∏i=1ndi!(2m)!∏i≤jmij!,

where $m_{2}={\sum \sum }_{i<j}m_{ij}$.

**Figure 1.**
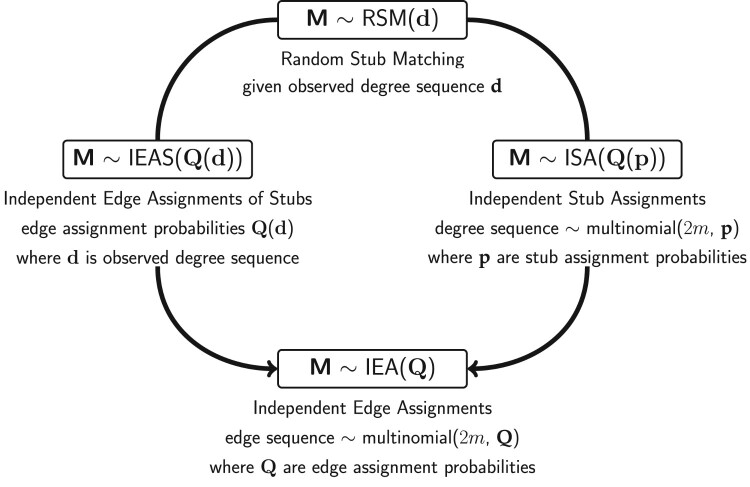
The presented two ways (IEAS and ISA) in which an approximate IEA model can be obtained from an RSM model.

There are two ways in which the distribution of M can be approximated with an IEA model [23]. These approximations are of particular interest since they simplify the derivation of several statistics use to infer structural local and global properties of a multigraph. We present these approximations in Figure 1 and describe them in more detail in the following.

A Bayesian version of the RSM model is obtained by assuming that the stubs are independently assigned to vertices according to a probability distribution $p=(p_{1},\ldots ,p_{n})$. The stub multiplicity sequence under independent stub assignments (ISA) is multinomially distributed with parameters 2m and p. This multinomial distribution can be viewed as a Bayesian model for the stub multiplicities and leads to independent edge assignments. Thus, by the Bayesian assumption, the RSM model is turned into a special IEA model with edge probability sequence Q defined as a function of p according to

(6) $$Q_{ij}=\left\{ \begin{array}{ll} p_{i}^{2} & \text{for }i=j\\ 2p_{i}p_{j} & \text{for }i<j. \end{array}\right.$$

The second way to get an approximate IEA model from an RSM model is to ignore the dependency between the edge assignments in the RSM model. The edge probability sequence Q=Q(d) of the RSM model is used to define a model with independent edge assignment of stubs (IEAS). Note that the IEAS model, like other IEA models, has (m+r−1m) different outcomes of M, while the RSM models are restricted to outcomes that are consistent with stub multiplicity sequence d only.

Throughout this paper, the following notations will be used for the models presented in this section. Independent edge assignment is denoted IEA(Q), random stub matching is denoted RSM(d), independent stub assignments is denoted ISA(p), and independent edge assignments of stubs is denoted IEAS(d).

## 3. Tests of a simple multigraph hypothesis

A simple multigraph hypothesis $H_{0}$ is defined as a fully specified $\mathrm{IEA}(Q_{0})$ which can be an $\mathrm{ISA}(p_{0})$ or an $\mathrm{IEAS}(d_{0})$ with $Q_{0}$ specified as a function of $d_{0}$ or $p_{0}$. The tests are performed using goodness of fit measures between the multiplicity sequence m of an observed multigraph and the expected multiplicity sequence according to $H_{0}$.

Asymptotic theory for likelihood ratios and goodness of fit statistics is given for instance by Andersen [1] and Cox and Hinkley [3]. The Pearson goodness of fit statistic is given by

(7) $$S_{0}=\underset{i\leq j}{\sum \sum }\frac{(m_{ij}-mQ_{0ij}{)}^{2}}{mQ_{0ij}}=\underset{i\leq j}{\sum \sum }\frac{m_{ij}^{2}}{mQ_{0ij}}-m,$$

which is asymptotically $\chi^{2}$-distributed with *df* = *r*−1 degrees of freedom, where r=(n+12), and if the multiplicity sequence is obtained according to IEA(Q) and the correct model $Q_{0}=Q$ is tested. We denote a random variable with this distribution $\chi_{r-1}^{2}$. Divergence statistics are used as goodness of fit statistics for instance by Frank *et al.* [5,6] and Kullback [15]. The divergence statistic is given by

(8) $$D_{0}=\underset{i\leq j}{\sum \sum }\frac{m_{ij}}{m}\log\frac{m_{ij}}{mQ_{0ij}},$$

and an asymptotic $\chi_{r-1}^{2}$-statistic can be obtained as

(9) $$A_{0}=2mD_{0},$$

[5,6,15]. For good asymptotic results, it is normally assumed that *m* is large and $mQ_{ij}$ is not too small (for instance $mQ_{ij}\geq 5$ and m≥5r). By approximation of the logarithm function it can be shown that $S_{0}\approx A_{0}$ for large *m*.

The critical region for the tests is taken as values of $S_{0}$ and $A_{0}$ above a critical value *cv* given by

(10) $$cv=df+2\sqrt{2df}=r-1+\sqrt{8(r-1)},$$

which has a significance level approximately equal to 5% given by $\alpha =P(\chi_{r-1}^{2}>cv)$.

The power functions

(11) $$P(S_{0}>cv)=1-\beta_{S_{0}}(Q)\;\mathrm{and}\;P(A_{0}>cv)=1-\beta_{A_{0}}(Q)$$

are calculated using the distributions of $S_{0}$ and $A_{0}$ when M is multinomially distributed with parameters *m* and Q, for $Q=Q_{0}$ and for $Q\neq Q_{0}$. Specifically, $S_{0}$ and $A_{0}$ are compared to $\chi_{r-1}^{2}$ via moments and cumulative distribution functions. For instance, the expected value of $S_{0}$ reveals how far from $E(\chi_{r-1}^{2})=r-1$ the distribution of $S_{0}$ is. This expected value is given by

(12) $$E(S_{0})=\underset{i\leq j}{\sum \sum }\frac{E(m_{ij}^{2})}{mQ_{0ij}}-m=\underset{i\leq j}{\sum \sum }\frac{Q_{ij}+(m-1)Q_{ij}^{2}}{Q_{0ij}}-m,$$

where $M_{ij}$ is binomially distributed with parameters *m* and $Q_{ij}$ so that

(13) $$E(M_{ij}^{2})=\mathrm{Var}(M_{ij})+[E(M_{ij}){]}^{2}=mQ_{ij}+m(m-1)Q_{ij}^{2}.$$

In particular, if $Q=Q_{0}$ so that $Q_{ij}=Q_{0ij}$ for i≤j, the null distribution of $S_{0}$ has expected value

(14) $$E(S_{0})=\underset{i\leq j}{\sum \sum }\left[1+(m-1)Q_{ij}\right]-m=r-1.$$

Under the ISA(p) model and $\mathrm{ISA}(p_{0})$ hypothesis, the expected value of $S_{0}$ is given as

(15) $$E(S_{0})=\frac{\sum_{i=1}^{n}L_{i}^{2}+{\left(\sum_{i=1}^{n}L_{i}\right)}^{2}}{2}-m+(m-1){\left(\sum_{i=1}^{n}L_{i}p_{i}\right)}^{2}$$

where $L_{i}=p_{i}/p_{0i}$ is the likelihood ratio for stub assignments. The derivation of this expected value is given in Appendix A.1. As seen, the variation of $E(S_{0})$ depends on $\sum_{i=1}^{n}L_{i}$, $\sum_{i=1}^{n}L_{i}^{2}$ and $\sum_{i=1}^{n}L_{i}p_{i}$. In particular, for a uniform $\mathrm{ISA}(p_{0})$ hypothesis where $p_{oi}=1/n$,

(16) $$E(S_{0})=\frac{n^{2}\sum_{i=1}^{n}p_{i}^{2}+n^{2}}{2}-m+(m-1)n^{2}{\left(\sum_{i=1}^{n}p_{i}^{2}\right)}^{2},$$

which by letting $s_{2}=\sum_{i=1}^{n}p_{i}^{2}$ can be simplified to

(17) $$E(S_{0})=m(n^{2}s_{2}^{2}-1)+\frac{n^{2}}{2}(1+s_{2}-2s_{2}^{2}).$$

From this we see that $E(S_{0})$ grows linearly with *m* having coefficients depending on *n* and $s_{2}$. By using

(18) $$E(S_{0})=s_{2}^{2}n^{2}(m-1)+s_{2}\frac{n^{2}}{2}+\frac{n^{2}}{2}-m$$

and $1/n\leq s_{2}\leq 1$, it follows that

(19) $$r-1\leq E(S_{0})\leq m(n^{2}-1).$$

We also note that if $p=p_{0}$ so that $p_{i}=p_{0i}$, the null distribution has

(20) E(S0)=n+n22−m+(m−1)=(n+12)−1=r−1

which is consistent with the result shown previously for $Q=Q_{0}$.

The expected value of $S_{0}$ can also be considered for the RSM(d) model when $H_{0}$ is $\mathrm{RSM}(d_{0})$ or $\mathrm{IEAS}(d_{0})$ since $Q_{0}$ of IEAS and RSM are identical. The moments of $M_{ij}$ under RSM are given by Shafie [23] as

(21) $$E(M_{ij})=mQ_{ij}\;\text{for }i\leq j,$$

and

(22) $$\mathrm{Var}(M_{ij})=\sigma_{ij}^{2}+\Delta_{ij}\;\text{for }i\leq j,$$

where $\sigma_{ij}^{2}=mQ_{ij}(1-Q_{ij})$ is the variance under IEA, and $\Delta_{ij}$ is the difference between the variances of $M_{ij}$ under RSM and IEA:

(23) $$\Delta_{ij}=m(m-1)(Q_{ijij}-Q_{ij}^{2}),$$

where

(24) $$Q_{ijij}=\left\{ \begin{array}{ll} Q_{ii}\left(\frac{\left(d_{i}-2)(d_{i}-3\right)}{\left(2m-2)(2m-3\right)}\right) & \text{for }i=j\\ Q_{ij}\left(\frac{2(d_{i}-1)(d_{j}-1)}{\left(2m-2)(2m-3\right)}\right) & \text{for }i<j. \end{array}\right.$$

A general expression for the expected value of $S_{0}$ under RSM is obtained as

(25) $$E(S_{0})=\underset{i\leq j}{\sum \sum }\frac{mQ_{ij}(1-Q_{ij})+\Delta_{ij}+m^{2}Q_{ij}^{2}}{mQ_{0ij}}-m.$$

The derivation of this expected value is given in Appendix A.2. For $Q=Q_{0}$, so that $Q_{ij}=Q_{0ij}$ for i≤j, this simplifies to

(26) $$E(S_{0})=\frac{\left(m-1)n(n-1\right)}{2m-3}.$$

This simplification is shown in detail in Appendix A.2 and implies that the expected value of the null distribution only depends on the number of vertices and edges. Using this expression we can now show for which values of *m* and *n* the expected value of $S_{0}$ under RSM is smaller than *r*−1, i.e.

(27) $$E(S_{0})=\frac{\left(m-1)n(n-1\right)}{2m-3}<(r-1)=\frac{n(n+1)}{2}-1.$$

Solving the inequality for *m* gives the following results:

(28) $$\begin{array}{rl} E(S_{0}) & <r-1\;\mathrm{for}\;m>\frac{n+6}{4},\\ E(S_{0}) & =r-1\;\mathrm{if}\;m=\frac{n+6}{4}\;\text{is integer},\\ E(S_{0}) & >r-1\;\mathrm{for}\;m<\frac{n+6}{4}. \end{array}$$

Note that the restriction 2m≥n imposed by no isolated vertices implies that $E(S_{0})>r-1$ only for some degenerate cases (*n* = 2, *m* = 1) and the extreme cases *n* = 3 or 4, and *m* = 2. Therefore, under RSM the null distribution of the test statistic $S_{0}$ has for all other cases an expected value below *r*−1, and its cumulative distribution function will tend to lie on or above that of $\chi_{r-1}^{2}$ for all practical useful cases. Exceptional cases with m<(n+6)/4 have so few stubs to be matched that they are unlikely to be useful in practice. Compare the requirement of large *m* needed for good $\chi^{2}$ asymptotic behaviors. Note however that the test statistics may not have asymptotic $\chi^{2}$-distributions under RSM due to dependency between edges.

Any test statistic *S*, like $S_{0}$ or $A_{0}$, can be approximated by an adjusted $\chi^{2}$-distribution which is useful for improving power calculations. Such approximations are given by

(29) $$S^{*}=\frac{\mu }{k}\chi_{k}^{2},$$

where μ=E(S). For any positive integer *k* the approximation $S^{*}$ has the same mean as *S* and a variance given by

(30) $$\mathrm{Var}(S^{*})=\frac{2\mu^{2}}{k}.$$

Two particular approximations $S^{\prime }$ and $S^{{}^{\prime \prime }}$ are given by $S^{*}$ for *k* chosen as the integer part of *μ* and for *k* = *r*−1, respectively. Their variances are

(31) $$\mathrm{Var}(S^{\prime })=\frac{2\mu^{2}}{\left\lfloor \mu \right\rfloor }\;\mathrm{and}\;\mathrm{Var}(S^{{}^{\prime \prime }})=\frac{2\mu^{2}}{r-1},$$

and the preferred approximation is the one with variance closest to $\mathrm{Var}(S)=\sigma^{2}$. Equivalently, the preferred adjusted $\chi^{2}$-distribution is the one with degrees of freedom closest to $2\mu^{2}/\sigma^{2}$.

## 4. Tests of a composite multigraph hypothesis

The composite multigraph hypothesis might be ISA for unknown p or IEAS for unknown d. The parameters have to be estimated from data m. These estimates are denoted $\hat{p}=\hat{p}(m)$ and $\hat{d}=\hat{d}(m)$, and they are related according to

(32) $$\hat{p}=\frac{\hat{d}}{2m},$$

where

(33) $${\hat{d}}_{i}=\sum_{j=1}^{n}(m_{ij}+m_{ji})=m_{i\cdot }+m_{\cdot i}\;\text{for }i=1,\ldots ,n,$$

and $m_{ij}=0$ for *i*>*j*. Thus, we have estimated sequences $\hat{Q}=({\hat{Q}}_{ij}:(i,j)\in R)$ in the two cases with composite ISA and IEAS hypotheses. Note that for ISA

(34) $${\hat{Q}}_{ij}=\left\{ \begin{array}{ll} {\hat{p}}_{i}^{2} & \text{for }i=j\\ 2{\hat{p}}_{i}{\hat{p}}_{j} & \text{for }i<j, \end{array}\right.$$

and for IEAS

(35) Q^ij={(d^i2)/(2m2)for i=jd^id^j/(2m2)for i<j.

The Pearson goodness of fit and divergence statistics are given as

(36) $$\hat{S}=\underset{i\leq j}{\sum \sum }\frac{(m_{ij}-m{\hat{Q}}_{ij}{)}^{2}}{m{\hat{Q}}_{ij}}=\underset{i\leq j}{\sum \sum }\frac{m_{ij}^{2}}{m{\hat{Q}}_{ij}}-m,$$

and

(37) $$\hat{D}=\underset{i\leq j}{\sum \sum }\frac{m_{ij}}{m}\log\frac{m_{ij}}{m{\hat{Q}}_{ij}}.$$

Here, $\hat{S}$ and $\hat{A}=2m\hat{D}$ are asymptotically χ(n2)2-distributed when the correct model is tested (this follows from the same logic and derivation as Equation (9)). Note that the number of degrees of freedom here is given as the difference in numbers of estimated free parameters without and with the hypothesis, i.e. df=(r−1)−(n−1)=r−n=(n2). The critical regions for these tests are given by values of $\hat{S}$ and $\hat{A}$ above a critical value *cv* which can be chosen as

(38) cv=df+22df=(n2)+8(n2)

to get a significance level close to 5% given by α=P(χ(n2)2>cv). The power functions $P(\hat{S}>cv)$ and $P(\hat{A}>cv)$ are functions of p or d depending on whether an ISA(p) or IEAS(d) model is considered. The error probabilities of false rejection and false non-rejection are denoted by *α* and *β*, respectively, and indexed by $\hat{S}$ and $\hat{A}$.

Similar to the test statistic approximations described in Section 3, $S^{\prime }$ and $S^{{}^{\prime \prime }}$ are here given by $S^{*}$ for *k* chosen as the integer part of *μ* and *r*−*n*, respectively. These approximations can be used as alternative test statistics provided the expected values of $\hat{S}$ and $\hat{A}$ are known. Formal expressions for the expected values are complicated to obtain due to that m is involved also via $\hat{Q}$ that depends on $\hat{d}$ which is determined by m. However, for our theoretical investigation we use complete enumerations of all outcomes of m and find the expected values and variances numerically. Under an RSM( d) model the estimated $\hat{d}$ is always (for any data m) equal to the d specified in the model which implies that

(39) $$E(\hat{S})=E(S_{0})=\frac{\left(m-1)n(n-1\right)}{2m-3},$$

as shown in Section 3 and Appendix A.2. The preferences between approximations to the test statistics and adjusted $\chi^{2}$-distribution are determined by comparing variances and degrees of freedom closest to $2\mu^{2}/\sigma^{2}$, as mentioned in Section 3.

## 5. Some test illustrations

### 5.1. Simple multigraph hypotheses against IEA models

**Figure 2.**
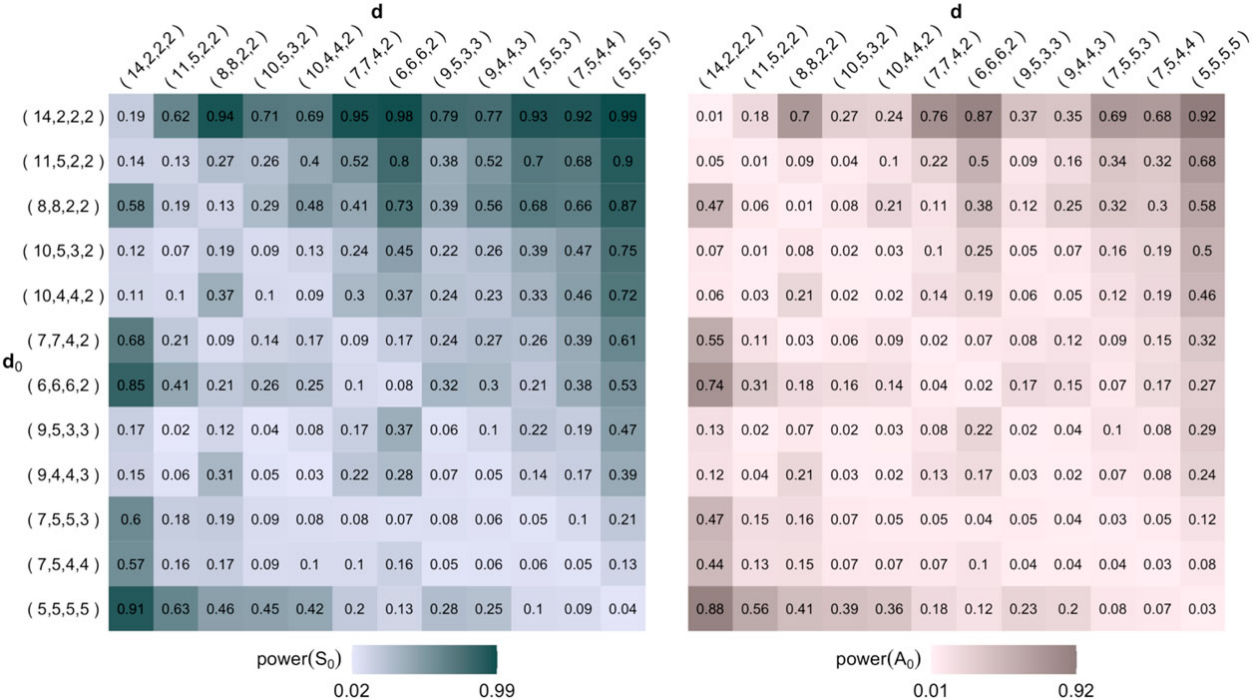
Power according to test statistics $S_{0}$ and $A_{0}$ when some simple IEAS( $d_{0}$) hypotheses are tested against IEAS( d) models for multigraphs with *n* = 4 and *m* = 10. The significance level for the asymptotic $\chi_{9}^{2}$-distribution is 0.04.

In this section, we simulate tests of IEA models, including both ways (IEAS and ISA) in which this model can be approximated by the RSM model (see Figure 1). We start by looking at multigraphs with 4 vertices and 10 edges and test IEAS( $d_{0}$) hypotheses against IEAS( d) models. The degree sequences are chosen to include both skew and flat (uniform and close to uniform) cases. The number of edge sites is here given by *r* = 10 and the test statistics $S_{0}$ and $A_{0}$ are thus asymptotically $\chi_{9}^{2}$-distributed when the correct model with $d_{0}=d$ is being tested. The critical value is *cv* = 17.49 and $\alpha =P(\chi_{9}^{2}>cv)=0.04$. The powers of these tests according to $S_{0}$ and $A_{0}$ are shown in Figure 2, where the degree sequences are ordered from skew to flat cases.

We first consider the diagonal cases $d_{0}=d$ in Figure 2, representing tests of correctly specified models. Generally for these cases, we note that the probabilities of false rejection are $\alpha_{A_{0}}=1-\beta_{A_{0}}<\alpha \leq 1-\beta_{S_{0}}=\alpha_{S_{0}}$, indicating a better performance of the statistic $A_{0}$. Specifically, for flat $d_{0}=d$, both statistics have significance levels equal or close to *α*, but for skew $d_{0}=d$, the significance level of $A_{0}$ is much below *α* and that of $S_{0}$ is much above *α*.

For majority of the off diagonal cases $d_{0}\neq d$ in Figure 2, we note that larger differences between the degree values in models and hypotheses result in powers being close or equal to one for both statistics. However, the inequalities between the two statistics persist indicating that their cumulative distribution functions can approach an asymptotic distribution from either below or above.

**Figure 3.**
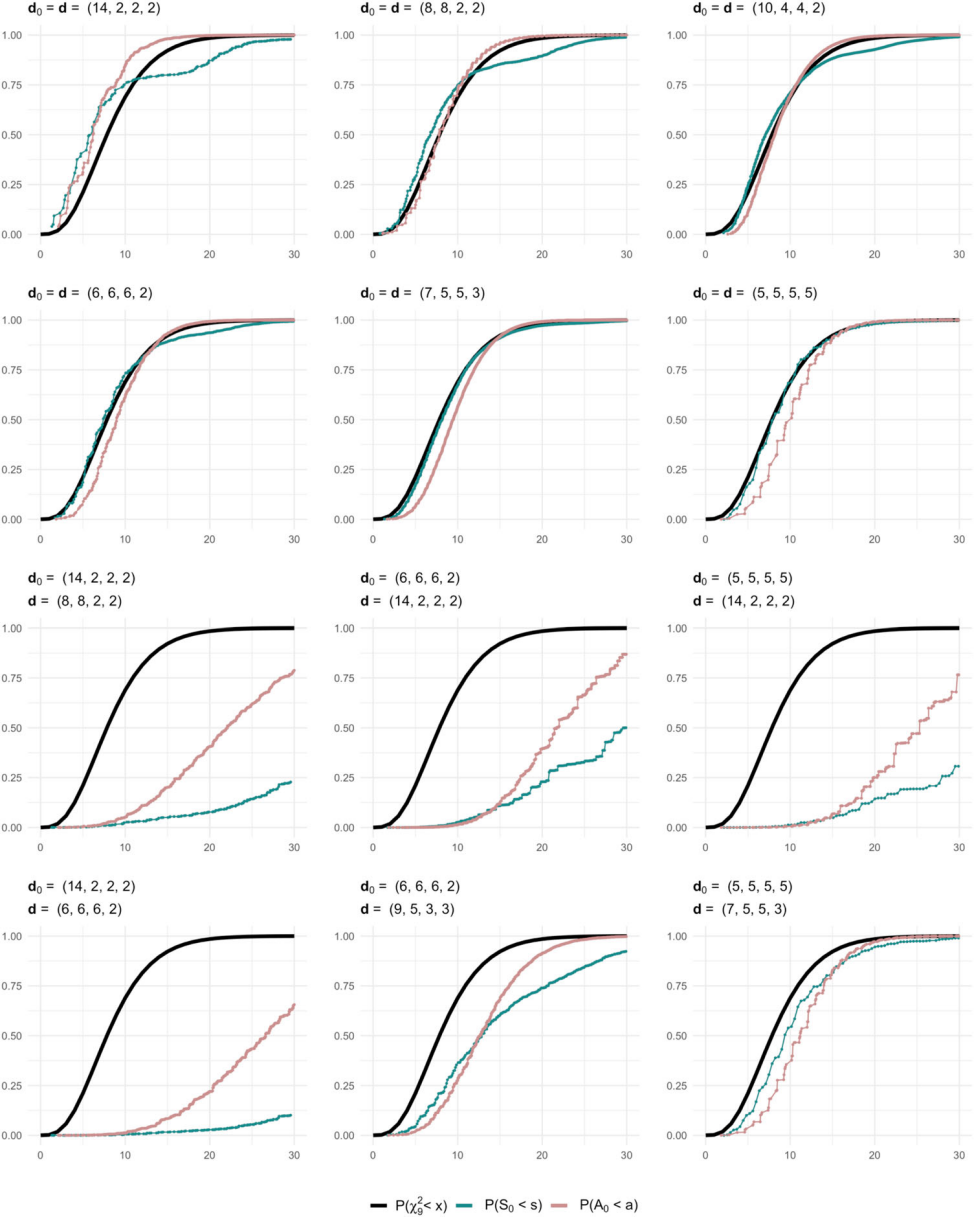
Null and non-null distributions of $S_{0}$ and $A_{0}$, and the $\chi_{9}^{2}$-distribution when some simple IEAS( $d_{0}$) hypotheses are tested against IEAS( d) models for multigraphs with *n* = 4 and *m* = 10.

To illustrate the fit of the distributions of the statistics $S_{0}$ and $A_{0}$ to $\chi_{9}^{2}$, their cumulative null and non-null distribution functions are shown in Figure 3. For flat $d_{0}=d$, the null distribution of $S_{0}$ almost coincides with that of $\chi_{9}^{2}$. For skew $d_{0}=d$, the null distributions of both statistics give poor fit to $\chi_{9}^{2}$-distribution. This poor fit is also noted for both flat and skew $d_{0}\neq d$ shown in Figure 3, with the exception of a slightly better fit to $\chi_{9}^{2}$-distribution when a flat or almost flat $d_{0}$ is tested against a flat or almost flat d, making it harder to detect the wrongly specified hypotheses for these cases. Overall, when $d_{0}\neq d$, both $S_{0}$ and $A_{0}$ have distributions that would be better approximated by $\chi^{2}$ with degrees of freedom chosen to be higher than *r*−1.

The convergence speed of the cumulative distribution functions of $S_{0}$ and $A_{0}$ is illustrated in Section 1 of supplementary material, where both flat and skew degree sequences are considered. The number of edges *m* increases as multiples of the chosen flat degree sequence d=(3,3,3,3) and skew degree sequence d=(6,2,2,2), when $d_{0}=d$ and $d_{0}\neq d$. We note that even for small *m*, the null distributions of both statistics are fairly well approximated by their asymptotic $\chi^{2}$-distribution. A similar investigation of the non-null distributions of $S_{0}$ and $A_{0}$ for flat and skew $d_{0}\neq d$ is shown in supplementary material where $d_{0}$ is kept fixed and d is varied. For both flat and skew $d_{0}$, the deviations between the non-null distributions of $S_{0}$ and $A_{0}$ and their asymptotic null distribution increase with the number of edges, and even for *m* = 12 this deviation is clearly notable. Thus, even for these rather small *m* = 6 and *m* = 12, it easy to detect simple hypotheses about false models. This is noted in particular when there are large differences between the degree values in models and hypotheses.

**Figure 4.**
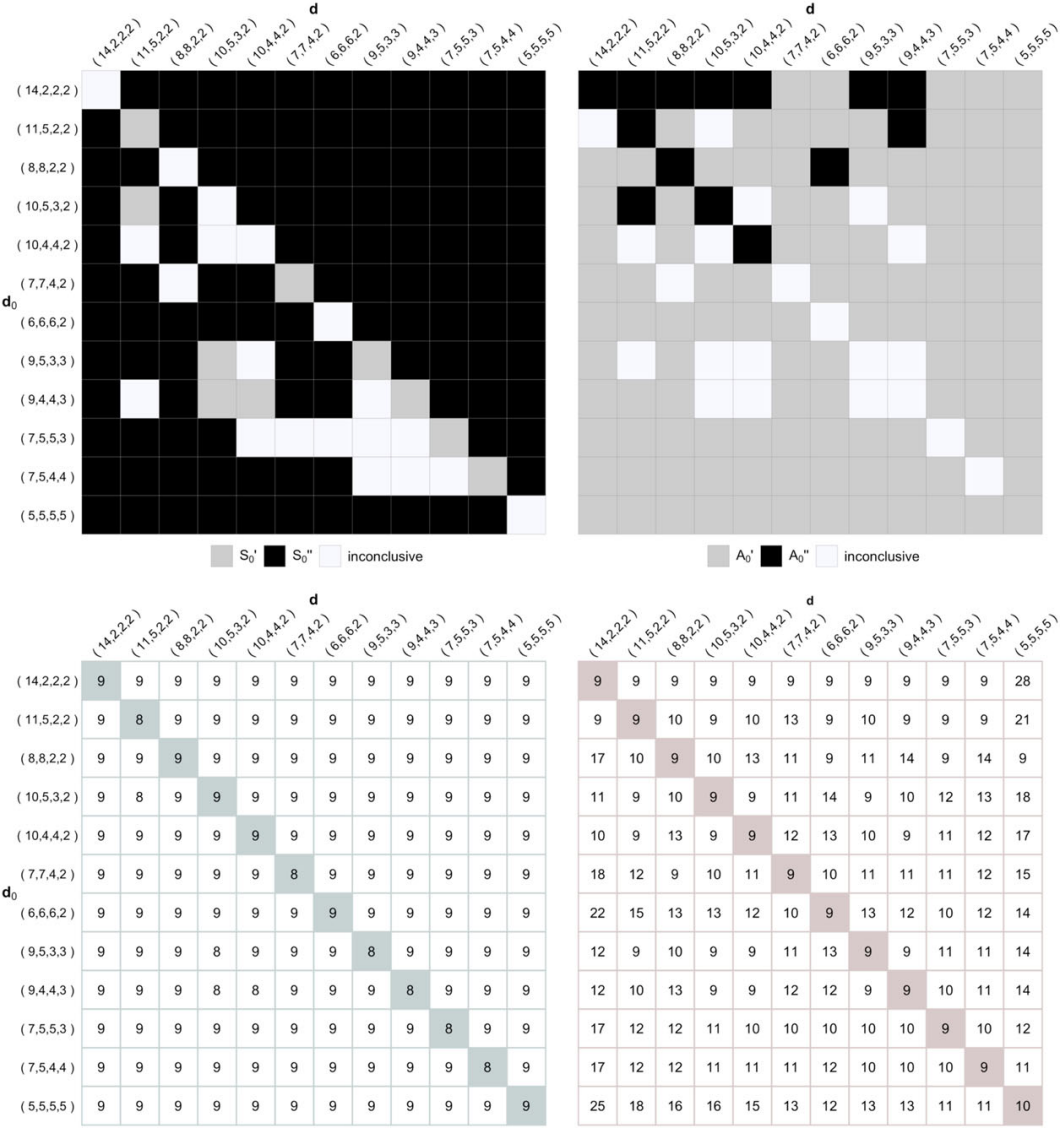
Preferred adjusted test statistics (top) and the degrees of freedom for preferred adjusted $\chi^{2}$-distribution (bottom) for $S_{0}$ (left) and $A_{0}$ (right), when some simple IEAS( $d_{0}$) hypotheses are tested against IEAS( d) models for multigraphs with *n* = 4 and *m* = 10.

In Figure 4, we illustrate how test statistics can be approximated by adjusted $\chi^{2}$-distributions. The approximated goodness of fit statistics are $S_{0}^{\prime }$ and $S_{0}^{{}^{\prime \prime }}$, and the approximated divergence statistics are $A_{0}^{\prime }$ and $A_{0}^{{}^{\prime \prime }}$, as presented in Section 3. These approximations are evaluated by comparing their variances to $\mathrm{Var}(S_{0})$ and $\mathrm{Var}(A_{0})$, and the best approximations are the ones with variances closest to $\mathrm{Var}(S_{0})$ and $\mathrm{Var}(A_{0})$, respectively (note that means of the test statistics and their respective approximations are always equal). We note the following tendencies in Figure 4. For majority of $S_{0}$ cases (top left), $S_{0}^{{}^{\prime \prime }}$ is preferred with the exception of testing correctly specified models as given in the diagonal $d_{0}=d$. For the majority of $A_{0}$ cases (top right), $A_{0}^{\prime }$ is preferred over $A_{0}^{{}^{\prime \prime }}$. The inconclusive cases are due the variances of adjusted statistics being equal.

Similarly, the preferred adjusted $\chi^{2}$-distributions for $S_{0}$ and $A_{0}$ are the ones having degrees of freedom closest to $2E(S_{0}{)}^{2}/\mathrm{Var}(S_{0})$ and $2E(A_{0}{)}^{2}/\mathrm{Var}(A_{0})$, respectively. The degrees of freedom for these preferred adjusted $\chi^{2}$-distributions are shown in the bottom row of Figure 4. For example, consider the test of the IEAS multigraph hypothesis with $d_{0}=(6,6,6,2)$ tested against the IEAS model with d=(8,8,2,2). The adjusted $\chi^{2}$-distribution for $S_{0}$ has *df* = *r*−1 = 9 since it is closer to $2E(S_{0}{)}^{2}/\mathrm{Var}(S_{0})=7.31$ than df=⌊μ⌋=13 (bottom left in Figure 4). The adjusted $\chi^{2}$-distribution for $A_{0}$ has df=⌊μ⌋=13 since it is closer to $2E(A_{0}{)}^{2}/\mathrm{Var}(A_{0})=18.19$ than *df* = *r*−1 = 9 (bottom right in Figure 4). We note that the majority of $S_{0}$ cases (bottom left), have correctly specified degrees of freedom of *r*−1, with a few exceptions. For the $A_{0}$ cases (bottom right), we note a much larger variation. The results here indicate that distributions are better approximated by $\chi^{2}$ with degrees of freedom chosen to be higher than *r*−1, in particular when $d_{0}\neq d$. This also means that the cumulative non-null distribution functions of $A_{0}$ approach the asymptotic $\chi^{2}$-distribution from below.

We now turn our attention to the second way in which an IEA model can be obtained and focus on ISA( $p_{0}$) hypotheses tested against ISA( p) models and consider tests of different multigraphs of the same size. The stub selection probability sequences are here chosen as the already considered degree sequences for the IEAS models divided by total number of edges, i.e. $p_{0}=d_{0}/2m$ and p=d/2m. Thus, we cover both skew and flat cases of stub selection probabilities. Similar figures as those for the IEAS models above are given in Section 1 of supplementary material and briefly summarize below.

The results from these tests are very similar to those for the IEAS models already presented, and these similarities are due to resemblances between the two models. Test powers follow the same patterns as those already discussed for IEAS models. For $p_{0}=p$, $\alpha_{S_{0}}$ and $\alpha_{A_{0}}$ are on opposite sides of α=0.04 but they are both close to *α* except for very skew cases. For the majority of cases with $p_{0}\neq p$, both test statistics have reasonable powers unless $p_{0}$ and p are too close. The fit of the cumulative distributions of the statistics $S_{0}$ and $A_{0}$ to that of $\chi_{9}^{2}$ when ISA( $p_{0}$) hypotheses are tested against ISA( p) models also show similar results as those for IEAS models: even for small *m*, there is a fairly good fit for all illustrated cases, including both flat and skew $p_{0}=p$, and $p_{0}\neq p$. Furthermore, the impact on the null and non-null distributions of $S_{0}$ and $A_{0}$ for skew and flat $p_{0}$ when *m* increases show that the convergence to the asymptotic distribution is rapid for null distributions of both statistics, and the deviations between the non-null distributions of both statistics and their asymptotic null distribution increase with *m*. The latter result implies that adjusted $\chi^{2}$-distributions should be used to approximate the non-null distributions. The results regarding these preferred adjusted test statistics and $\chi^{2}$-distributions are also consistent with those already presented for the IEAS models. For the majority of $S_{0}$ cases, $S_{0}^{{}^{\prime \prime }}$ is preferred and have correctly specified degrees of freedom of *r*−1. For the majority of $A_{0}$ cases, $A_{0}^{\prime }$ is preferred over $A_{0}^{{}^{\prime \prime }}$, and the distributions are better approximated by $\chi^{2}$ with degrees of freedom chosen to be higher than *r*−1.

### 5.2. Simple multigraph hypotheses against RSM models

When performing tests of IEA models, multigraphs are known to have multiplicity sequences that are multinomially distributed, which implies that the distributions of the test statistics $S_{0}$ and $A_{0}$ are asymptotically $\chi^{2}$-distributed when the correct model is being tested. However, for RSM models, there is dependence between edges, and the distributions of $S_{0}$ and $A_{0}$ are unknown. In this section, we illustrate some of the consequences of using the previously described tests of simple hypotheses against a false IEA model, when the true model is RSM. Here, both IEAS( $d_{0}$) and ISA( $p_{0}$) hypotheses are tested for flat and skew $d_{0}$ and $p_{0}$. The true model is RSM( d) so that only non-null distributions of $S_{0}$ and $A_{0}$ are considered. Note that the restraint of number of multigraphs given degree sequence reduces the outcomes space for edge multiplicity sequences, allowing us to test multigraphs of larger size with respect to number of edges.

Multigraphs with 4 vertices and 30 edges are considered and all simulation outputs are shown in supplementary material Section 2. The powers of these tests according to $S_{0}$ and $A_{0}$ show the following. For the diagonal cases where $d_{0}=d$, both $\alpha_{S_{0}}$ and $\alpha_{A_{0}}$ are below α=0.04 implying that it is difficult to detect hypotheses about wrong models. This holds in particular for $A_{0}$ as the test statistic, and for $S_{0}$ when testing ISA hypotheses. For cases with $d_{0}\neq d$ and $p_{0}\neq d_{0}/2m$, both test statistics have very good or reasonable powers in the majority of cases. This holds if d is not too close to $d_{0}$, that is when testing skew against flat degree sequences, and vice versa, and is a consequence of similarities between IEAS and ISA models for large *m*. This is further illustrated in figures where the fit of the non-null distributions of the statistics $S_{0}$ and $A_{0}$ to that of $\chi_{9}^{2}$ are shown for some selected cases. We note similar trends as those for IEA models considered in Section 5.1; it is generally difficult to detect differences between how the models RSM, IEAS and ISA effect the test statistics. The similarity between modeled and hypothetical degree sequences determine the goodness of fits, and whether hypotheses about wrong models can be detected or not.

Further in Section 2 of supplementary material, non-null distributions of $S_{0}$ and $A_{0}$ for some RSM( d) models are illustrated where *m* increases as multiples of different specified d. This includes both IEAS hypotheses with flat $d_{0}$, and IEAS hypotheses with skew $d_{0}$. The following can be noted and summarized. When $d_{0}=d$, the non-null distributions of both $S_{0}$ and $A_{0}$ lie above the asymptotic null distribution. We see that as *m* increases, these distributions still lie above the asymptotic null distribution, and a $\chi^{2}$-distribution with lower degrees of freedom provides a better approximate to these distributions. For cases with $d_{0}\neq d$, the non-null distributions of both statistics move further away from the asymptotic null distribution as *m* increases. This implies a need to use adjusted $\chi^{2}$-distributions for better fit.

Illustration of how increasing *m* affects the fit between the non-null distributions of $S_{0}$ and $A_{0}$ and the asymptotic null distribution for ISA hypotheses with flat and skew $p_{0}$ are almost identical to those presented for IEAS hypotheses.

Looking at how how test statistics can be approximated by adjusted $\chi^{2}$-distributions for IEAS and ISA hypotheses tested against RSM models reveal the following tendencies: in almost all cases when $d_{0}\neq d$, the preferred adjusted test statistic is $S_{0}^{\prime }$ and $A_{0}^{\prime }$ as they have variances closest to $\mathrm{Var}(S_{0})$ and $\mathrm{Var}(A_{0})$, respectively. For the majority of diagonal cases $d_{0}\neq d$, $A_{0}^{{}^{\prime \prime }}$ is the preferred option, while the results are varying for statistic $S_{0}$. We also note large discrepancies between the adjusted $\chi^{2}$-distributions of the two test statistics. In particular for $d_{0}=d$, the adjusted $\chi^{2}$-distribution for $S_{0}$ seems to be closer to *r*−*n* rather than *r*−1 degrees of freedom under RSM. This is also supported by the expected value of $S_{0}$ which according to the result in Section 3 is (m−1)n(n−1)/(2m−3) which is about r−n=n(n−1)/2. As these cases represent non-null distributions, we would need much higher discrepancies between the degrees of freedom to the $\chi^{2}$ distributions in order to detect hypotheses about wrong models. For the off diagonal cases $d_{0}\neq d$ also representing non-null distributions, these discrepancies are much more evident (in particular for $A_{0}$), making it easy to detect these hypotheses about wrong models. We also note that it is easier to detect wrongly specified IEA hypotheses with skew $d_{0}$ tested against d, than wrongly specified ones with flat $d_{0}$ tested against d.

Note that we in in this section only considered the consequences of replacing IEA models with RSM models, but only tested IEA hypotheses. We provide a discussion and suggestion for the extension of testing RSM hypotheses in Section 6 of this paper and will not pursue details of this test further here.

### 5.3. Composite multigraph hypotheses against IEA models

Consider composite IEAS and ISA hypotheses against IEAS( d) and ISA( p) models where p=d/2m, for multigraphs with 4 vertices and 10 edges. When testing IEAS models, the composite IEAS hypotheses include the correct model and when testing ISA models, the composite ISA hypotheses include the correct model. For these cases, the probabilities of false rejection according to $\hat{S}$ and $\hat{A}$ are given in Section 3 of supplementary material.

When testing composite IEAS hypotheses against IEAS models, both $\alpha_{\hat{S}}$ and $\alpha_{\hat{A}}$ for flat d are close or equal to α=0.04. For skew d, $\alpha_{\hat{S}}$ remains close or equal to *α* while $\alpha_{\hat{A}}$ is much below. If the composite ISA hypothesis is instead tested against the IEAS( d) model, the powers of $\hat{S}$ are much below that of $\alpha_{\hat{S}}$ and the powers of $\hat{A}$ almost equal to the values $\alpha_{\hat{A}}$. Thus, both statistics have very poor powers of detecting differences between composite ISA and IEAS hypotheses, and these poor powers are due to the resemblances between the two models.

Similar tendencies are also evident when composite ISA and IEAS hypotheses are tested against ISA( p) models. However, note that when testing composite IEAS hypotheses against ISA models, $\alpha_{\hat{S}}$ are greater or equal to *α*, implying it is marginally easier to detect tests of the wrongly specified IEAS models against ISA models, than detecting tests of the wrongly specified ISA models against IEAS models.

Given the similarities between the two IEA models, we only consider composite ISA and IEAS hypotheses tested against IEAS models in the study of cumulative distributions of the test statistics which are shown in Section 3 of supplementary material. The fit of the distributions of $\hat{S}$ and $\hat{A}$ to that of $\chi_{6}^{2}$ show the following. For the very skew and very flat d, there are larger deviations from $\chi_{6}^{2}$. Moreover, for skew d, both $\hat{S}$ and $\hat{A}$ have distributions that would be better approximated by $\chi^{2}$ with lower degrees of freedom chosen, while for flat d, the distributions of the test statistics would be better approximated by $\chi^{2}$ with higher degrees of freedom chosen.

Studying the null and non-null distributions of $\hat{S}$ and $\hat{A}$ for some IEAS( d) models with flat and skew d, we note the following when *m* increases as multiples of the specified d. The convergence of the null and non-null distributions for flat d is rapid towards the asymptotic distribution, while the convergence of the distributions for skew d is slower for both statistics. Thus, for small and large *m*, it is difficult to detect differences between composite ISA and IEAS hypotheses.

By looking at how test statistics can be approximated by adjusted $\chi^{2}$-distributions for composite hypotheses tested against IEAS( d) and ISA( p) models (where p=d/2m) we note the following (see Section 3 in supplementary material). The preferred statistics ${\hat{S}}^{\prime }$, ${\hat{S}}^{{}^{\prime \prime }}$, ${\hat{A}}^{\prime }$ and ${\hat{A}}^{{}^{\prime \prime }}$ vary in different cases so no clear tendency can be noted. For majority of $\hat{A}$ cases, the adjusted $\chi^{2}$-distributions have correctly specified degrees of freedom of *r*−*n*, with the exceptions of the most skew and the most flat cases which approach the asymptotic $\chi^{2}$-distribution from below. The flat cases for statistic $\hat{S}$ also have approximately correct specified degrees of freedom while the skew cases have lower degrees of freedom than *r*−*n*, showing that the cumulative distribution functions of $\hat{S}$ approach the asymptotic distribution from above.

### 5.4. Composite multigraph hypotheses against RSM models

In this section we illustrate some of the consequences of using previously described tests of composite hypotheses against a false IEA model, when the true model is RSM. The output from these simulations are given in supplementary material Section 4, where IEAS( d) and ISA( p), with p=d/2m, are tested against RSM( d) models. We consider multigraphs with 4 vertices and 30 edges. The note poor powers according to $\hat{S}$ and $\hat{A}$ of rejecting IEAS and ISA when RSM is true. We see that $\alpha_{\hat{S}}$ is close to α=0.04 for all cases shown, while $\alpha_{\hat{A}}$ moves from being below *α* for skew d to being greater than $\alpha_{\hat{A}}$ for flat d. Thus, it is slightly easier to detect wrongly specified models for flat d when using test statistic $\hat{A}$.

To illustrate the fit of the distributions of the statistics $\hat{S}$ and $\hat{A}$ to that of $\chi_{6}^{2}$, we look at their cumulative distribution functions. For all cases, there is a reasonably good fit to $\chi_{6}^{2}$, indicating the variances of both test statistics are roughly twice their expected values which are equal to 6. Thus, the approximations of test statistics are mostly unnecessary for this rather large *m*. This is further noted when looking at the effects of increasing *m* on the non-null distributions of $\hat{S}$ and $\hat{A}$ for some RSM( d) models with flat and skew d, as illustrated in Section 4 of supplementary material. For all cases we see that these distributions are very close to the asymptotic null distribution. Further, the effect from increasing *m* on the non-null distributions is small.

## 6. Summary of test results

We summarize the main results from the tests performed in Section 5 in light of the problems and questions posed in Section 1. The convergence of the null distributions of *S* and *A* to their asymptotic $\chi^{2}$-distributions is rapid and even for small number of edges *m*, a good fit is seen between the null distributions and the asymptotic $\chi^{2}$-distribution. In other words, the asymptotic behavior of the test statistics is such that it can produce reliable results even for small multigraphs. This holds true for testing simple as well as composite hypotheses with different asymptotic distributions. Moreover, the influence of RSM on both test statistics is substantial for small number of edges and implies a shift of their distributions towards smaller values compared to what holds true for the null distributions under IEA. As the number of edges increases, the non-null distributions of both statistics move further away from the asymptotic null distribution implying a need to use adjusted $\chi^{2}$-distributions for better fit. Tests of RSM can be made by critical regions for m, but *S* and *A* cannot distinguish RSM from IEA. The non-null distributions of *S* and *A* needed for determining power can be well approximated by adjusted $\chi^{2}$-distributions and it is possible to judge how powers depend on the parameters of the IEA models.

For the simple IEA hypotheses tested against IEAS, ISA and RSM models in Sections 5.1 and 5.2, we note the following. For cases when flat d or p is tested against skew d or p (or vice versa), both statistics have good powers of rejecting a simple hypothesis about a false model. The non-null distributions of $S_{0}$ and $A_{0}$ needed for determining power can be well approximated by presented adjusted $\chi^{2}$-distributions.

For composite IEAS or ISA hypotheses including the correct model, the following results are noted from the tests performed in Section 5.3. The null distributions of $\hat{S}$ and $\hat{A}$ converge faster to their asymptotic $\chi^{2}$-distributions for flat d or p than for skew d or p, but even for rather small *m*, there is a good fit between these distributions and their asymptotic $\chi^{2}$-distributions. However, both statistics have very poor powers of detecting differences between IEAS and ISA hypotheses for small as well as for large *m*. From the tests in Section 5.4, it can be concluded that no matter the size of *m*, it is difficult to detect a false composite hypothesis under an RSM model, just as it is difficult to detect false composite hypotheses under IEA models.

The general trends regarding error probabilities *α* and *β* with respect to the two test statistics *S* and *A* are summarized in Table. 1 for all tests performed. When testing correctly specified simple IEA hypotheses against IEA models with $d_{0}=d$, $A_{0}$ gives lower probabilities of false positives *α*, especially for skew d. However, for all other simple tests in which $d_{0}\neq d$, the probabilities of false negatives *β* are higher for $A_{0}$ than for $S_{0}$. When simple IEA hypotheses are tested against RSM models, we get varying results and while most of these tests have good powers using both statistics, there are cases where a general trend cannot be determined.

**Table 1.** Summary of error probabilities according to $S_{0}$ and $A_{0}$ when simple IEAS( $d_{0}$) or ISA( $d_{0}/2m$) hypotheses, and according to $\hat{S}$ and $\hat{A}$ when composite IEAS and ISA hypotheses, are tested against IEAS( d), ISA( d/2m) or RSM( d) models.

		Simple IEAS( $d_{0}$) hypothesis			Composite hypothesis	
	Model	$d_{0}=d$	Flat $d_{0}\neq d$	Skew $d_{0}\neq d$	IEAS	ISA
IEAS	Flat d	$\alpha_{S_{0}}>\alpha_{A_{0}}$	$\beta_{S_{0}}<\beta_{A_{0}}$	$\beta_{S_{0}}<\beta_{A_{0}}$	$\alpha_{\hat{S}}\leq \alpha_{\hat{A}}$	$\beta_{\hat{S}}>\beta_{\hat{A}}$
	Skew d	$\alpha_{S_{0}}>\alpha_{A_{0}}$	$\beta_{S_{0}}<\beta_{A_{0}}$	$\beta_{S_{0}}<\beta_{A_{0}}$	$\alpha_{\hat{S}}>\alpha_{\hat{A}}$	$\beta_{\hat{S}}\geq \beta_{\hat{A}}$
		Simple ISA( $d_{0}/2m$) hypothesis			Composite hypothesis	
		$d_{0}=d$	Flat $d_{0}\neq d$	Skew $d_{0}\neq d$	IEAS	ISA
ISA	Flat d	$\alpha_{S_{0}}\geq \alpha_{A_{0}}$	$\beta_{S_{0}}\leq \beta_{A_{0}}$	$\beta_{S_{0}}<\beta_{A_{0}}$	inconclusive	$\alpha_{\hat{S}}\leq \alpha_{\hat{A}}$
	Skew d	$\alpha_{S_{0}}>\alpha_{A_{0}}$	$\beta_{S_{0}}\leq \beta_{A_{0}}$	$\beta_{S_{0}}<\beta_{A_{0}}$	$\beta_{\hat{S}}<\beta_{\hat{A}}$	$\alpha_{\hat{S}}>\alpha_{\hat{A}}$
		Simple IEAS( $d_{0}$) or ISA( $d_{0}/2m$) hypothesis			Composite hypothesis	
		$d_{0}=d$	Flat $d_{0}\neq d$	Skew $d_{0}\neq d$	IEAS	ISA
RSM	Flat d	$\beta_{S_{0}}\geq \beta_{A_{0}}$	Inconclusive	$\beta_{S_{0}}=\beta_{A_{0}}$	$\beta_{\hat{S}}>\beta_{\hat{A}}$	$\beta_{\hat{S}}>\beta_{\hat{A}}$
	Skew d	$\beta_{S_{0}}\leq \beta_{A_{0}}$	$\beta_{S_{0}}=\beta_{A_{0}}$	$\beta_{S_{0}}<\beta_{A_{0}}$	$\beta_{\hat{S}}\geq \beta_{\hat{A}}$	$\beta_{\hat{S}}>\beta_{\hat{A}}$

For the composite tests, the error probabilities according to $\hat{S}$ and $\hat{A}$ in Table 1 can be summarized as follows. For flat d, the probabilities of false rejection are lower for $\hat{S}$ than $\hat{A}$, while the opposite holds for skew d. The probabilities of failed rejection of incorrectly specified hypotheses are higher for IEAS models and not possible to determine for ISA models (can be either higher or lower). However, for incorrectly specified IEA hypotheses tested against RSM models, we note consistently better powers when $\hat{A}$ is used, for both skew and flat d. For RSM models, this is due to better asymptotic behavior since tests are performed on multigraphs with three times the number of edges as those for IEA models.

## 7. Applications

### 7.1. Outline of included real world examples

Local and global structural features of directly observed or aggregated multigraphs can be analyzed by applying the probability models presented in Section 2. However, statistics under the IEA model are far easier to apply since explicit formulas for their moments are derived in [22,23]. Some of these closed expressions are missing for the RSM model due to the combinatorial complexity it entails. Thus, testing the fit of the RSM approximations is important as it reveals the potential to apply statistics under the IEA models instead. Examples of mentioned statistics include number of loops which can be used to analyze homophily (i.e. higher likelihood to connect to those with similar vertex attributes) and statistics related to edge multiplicity counts to analyze multiplexity (i.e. edge entrainment for networks with multiple types of edges). Such statistics and their applications are presented in [22,23,25].

Below, we provide two applications in which aggregated multigraphs are used to test whether the observed data follows IEA approximations of the RSM model. By comparing the models and degree sequences to those simulated in earlier sections, our analysis can be guided and we can conclude whether test results can be trusted. However, given the uncertainty connected to the test results for composite multigraph hypotheses shown in Section 4, we only focus on simple null hypotheses specified for the tests performed. Non-rejection of the null implies that the approximations fit the data, thus implying that above mentioned statistics can be used to further analyze the observed network.

### 7.2. Florentine family networks

**Figure 5.**
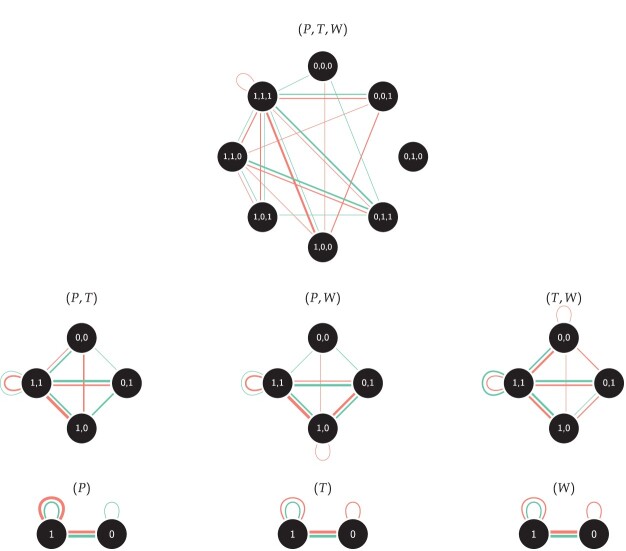
Aggregated multigraphs of the 16 Florentine families with marital (red) and financial (green) relations moving between and within categories based on vertex attributes political (*P*), social (*T*) and economic (*W*) influence. Thicker lines indicate higher edge multiplicity counts.

The first considered application is the well known network data on renaissance Florence families and how these families strategically formed alliances with each other to obtain a more powerful and important position in society [10,19]. In this subset of the original data set, we have 15 financial (*F*) and 20 marital (*M*) undirected ties between pairs of 16 families, together with three attributes observed on each family measuring economic, politic and social influence, respectively: net wealth in thousands of lira in 1427 (*W*), number of priorates i.e. number of seats on the Civic Council between 1282 and 1344 (*P*), the number of business and marriage ties in the total network data set consisting of the 116 families (*T*). Following the multigraph application in [22], we use binarized values to reflect weak (= 0) and strong (= 1) influences based on each attribute, thus simplifying the multigraph aggregations. Multigraphs on 2, 4 or 8 vertices can then be aggregated based on single or combined vertex attributes, as shown in Figure 5. The data set and edgelists for the aggregated multigraphs are given in Section 5.1 of the supplementary material (for more details regarding multigraph aggregations, see [22]).

The goodness of fit tests for all considered multigraphs in Figure 5 are summarized in Table 2. The *p*-values for testing whether there is a significant difference between observed and expected edge multiplicity values according to the two approximate IEA models are presented. These two approximations of the RSM model are IEAS( $d_{0}$) and ISA( $d_{0}/2m$) where $d_{0}$ is observed degree sequence. The tests where the null is rejected are shaded since they indicate that the model approximations, thus also the model statistics, cannot be used to further analyze structural properties. For these cases, adjusted test statistics and $\chi^{2}$-distributions presented in this paper can be considered for performing a power analysis.

**Table 2.** *P*-values when testing if there is a significant difference between observed and expected edge multiplicity values according to IEAS( $d_{0}$) and ISA( $d_{0}/2m$) models in the Florentine multigraphs aggregated based on single or combined binary vertex attributes representing political (*P*), social (*T*) and economic (*W*) influence (see Figure 5).

		Marital ties				Financial ties			
Multigraph	df	$d_{0}$		IEAS( $d_{0}$)	ISA( $d_{0}/2m$)	$d_{0}$		IEAS( $d_{0}$)	ISA( $d_{0}/2m$)
$m_{PTW}$	35	(13,9,7,4,3,3,1,0)	$S_{0}$	0.998	0.997	(9,8,5,3,2,2,1,0)	$S_{0}$	1.000	0.999
			$A_{0}$	0.988	0.983		$A_{0}$	0.998	0.996
$m_{PT}$	9	(20,12,5,3)	$S_{0}$	0.553	0.504	(14,8,4,4)	$S_{0}$	0.857	0.800
			$A_{0}$	0.060	0.050		$A_{0}$	0.497	0.422
$m_{PW}$	9	(16,16,7,1)	$S_{0}$	0.616	0.600	(12,10,6,2)	$S_{0}$	0.906	0.860
			$A_{0}$	0.090	0.078		$A_{0}$	0.494	0.431
$m_{TW}$	9	(16,10,7,7)	$S_{0}$	0.957	0.934	(17,5,5,3)	$S_{0}$	0.930	0.885
			$A_{0}$	0.366	0.303		$A_{0}$	0.252	0.200
$m_{P}$	2	(32,8)	$S_{0}$	0.004	0.004	(18,12)	$S_{0}$	0.314	0.300
			$A_{0}$	0.000	0.000		$A_{0}$	0.002	0.002
$m_{T}$	2	(23,17)	$S_{0}$	0.037	0.047	(22,8)	$S_{0}$	0.000	0.046
			$A_{0}$	0.000	0.000		$A_{0}$	0.000	0.000
$m_{W}$	2	(23,17)	$S_{0}$	0.239	0.242	(22,8)	$S_{0}$	0.046	0.045
			$A_{0}$	0.000	0.000		$A_{0}$	0.000	0.000

Note: Cases where the null is rejected based on a significance level of 0.05 are shaded.

We note the following from Table 2. First, observed degree sequences for all aggregated multigraphs are skewed. Following our summary in Table 1, we can therefore conclude te Pearson statistic $S_{0}$ is more reliable in terms of minimizing the error probabilities *α* and *β*. Second, with a few exceptions, both statistics and approximations yield the same test results.

**Figure 6.**
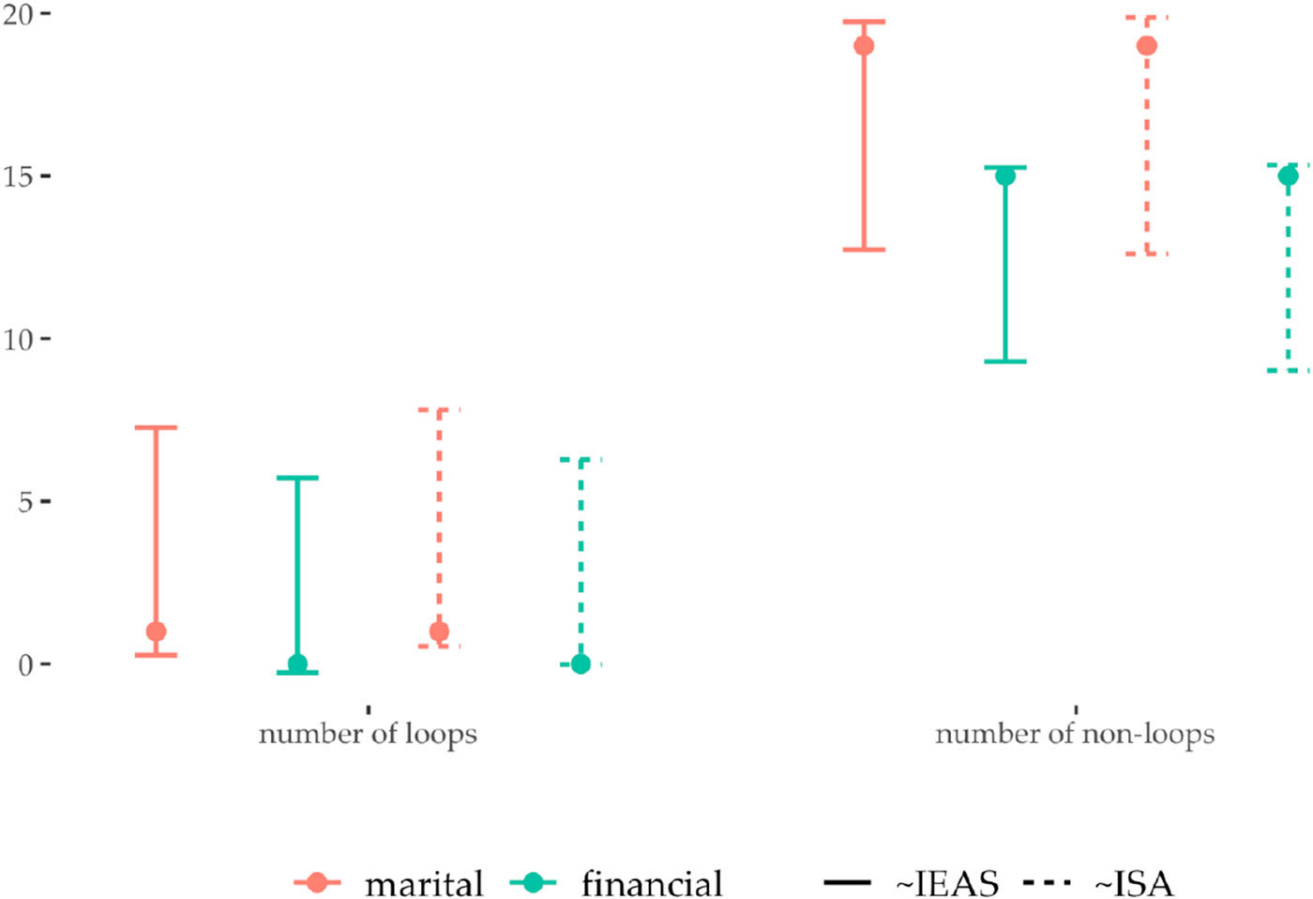
Approximate 95% interval estimates for the statistics number of loops and number of non-loops in the Florentine multigraph $m_{PTW}$ shown in Figure 5. These intervals are calculated for the IEAS( $d_{0}$) and the ISA( $d_{0}/2m$) model where $d_{0}$ is the observed degree sequence. The observed counts of number of loops and non-loops are given as filled circles in each interval.

In order to exemplify how non-rejection of the specified null can facilitate the structural analysis, we focus on the networks aggregated based on all three vertex attributes. When testing the fit of the IEA models on these multigraphs, we get the highest *p*-values implying the strongest evidence for the null such that we fail to reject it. Thus, there is not a significant difference between the observed and the expected edge multiplicity sequence based on the two IEA models. Statistics derived under these models can thus be used to analyze the structure of these multigraphs. To illustrate this, we focus on the following two statistics: number of loops denoted $M_{1}$ and number of non-loops denoted $M_{2}$. Because these statistics have the linear relationships $M_{2}=m-M_{1}$, their expected values are given by

(40) $$E(M_{1})=m\sum_{i=1}^{n}Q_{ii}\;\mathrm{and}\;E(M_{2})=m-E(M_{1}),$$

and they have a common variance given by

(41) $$\mathrm{Var}(M_{1})=\mathrm{Var}(M_{2})=\sum_{i=1}^{n}\sum_{j=1}^{n}\mathrm{Cov}(M_{ii},M_{jj})=m\left[\sum_{i=1}^{n}Q_{ii}(1-Q_{ii})-\underset{i\leq j}{\sum \sum }Q_{ii}Q_{jj}\right],$$

under the IEA model [22]. These statistics can be used to analyze homophily and heterophily, i.e. stronger tendency to connect to those with similar attributes and to those with dissimilar attributes Figure 6 illustrates approximate 95% interval estimates for $M_{1}$ and $M_{2}$ given by $\hat{E}\pm 2\sqrt{\hat{\mathrm{Var}}}$. These are shown for the two relations in the multigraphs $m_{PTW}$ and when applying the IEAS( $d_{0}$) and ISA( $d_{0}/2m$) model with observed degree sequence $d_{0}$. The observed counts fall within each interval and are given as filled circles.

The following can be concluded from Figure 6. Given the similarity between the IEAS and ISA model, the intervals are almost perfectly overlapping. We observe fewer homophilous ties than expected under models, while the number of non-loops indicates a higher number of heterophilous ties than expected. Thus, we can conclude that there is a higher propensity to connect to those with dissimilar attributes in this multigraph. We also not that marriage and finance intervals for $M_{1}$ and those for $M_{2}$ are overlapping. This could be an indicator that there is covariation between the two relation such that an analysis of multiplexity or edge entrainment should be considered as a next step in the structural analysis. How to perform such multiplexity analysis using a multigraph representation is shown in [25].

### 7.3. Friendship networks in a Dutch school class

**Figure 7.**
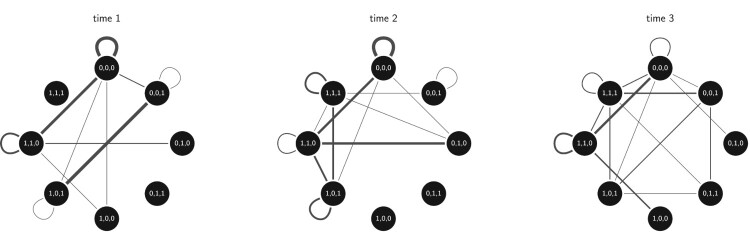
Aggregated multigraphs of friendship networks in a Dutch class over three time periods. The aggregations are based on combined binary attributes representing delinquent behavior, smoking and gender. Thicker lines indicate higher edge multiplicity counts.

The second example is performed using a longitudinal friendship network in a Dutch school class collected and applied by [11,12]. Here, strong and symmetrized friendship ties, together with constant and changing actor covariates on 26 students, were studied over their first year at a secondary school and at four time points at intervals. Complete observations on all attribute variables were only collected for three of the time periods and we consider these three in our application here. The two changing covariates are 'delinquent behavior' and 'drinking alcohol', both binarized to reflect if the pupils are never or at least once part of such behavior. These attributes together with the third constant covariate 'sex', were used to create the multigraphs on 8 vertices shown in Figure 7. The data set and edgelists for the aggregated multigraphs are given in Section 5.2 of the supplementary material.

We test if there is a significant difference between observed and expected the edge multiplicities given the IEAS( $d_{0}$) and ISA( $d_{0}/2m$) where $d_{0}$ is replaced by the observed degree sequence which we note is skewed for all cases. Table  3 summarizes these tests and as previous example, cases where we find significant difference are shaded implying we reject the null models. We note that this occurs for all tested multigraphs such that the statistics under the IEA model cannot be used to analyze these multigraphs further.

**Table 3.** *P*-values according $S_{0}$ and $A_{0}$ when simple IEAS( $d_{0}$) or ISA( $d_{0}/2m$) hypotheses, and according to $\hat{S}$ and $\hat{A}$ when composite IEAS and ISA hypotheses, are tested against IEAS( d), ISA( d/2m) or RSM( d) models.

Multigraph	df	$d_{0}$		IEAS( $d_{0}$)	ISA( $d_{0}/2m$)
time 1	35	(24,19,11,8,2,2,0,0)	*S*	0.004	0.008
			*A*	0.000	0.000
time 2	35	(21,19,17,9,7,3,0,0)	*S*	0.002	0.005
			*A*	0.001	0.002
time 3	35	(15,14,13,8,6,5,4,1)	*S*	0.000	0.000
			*A*	0.000	0.000

Note: Cases where the null is rejected based on a significance level of 0.05 are shaded.

However, another conclusion based on these test results in Table  3 can be reached. The *p*-values are decreasing over time implying an increased shift away from the null distributions. This could be due to two things. First, we may have a correctly specified null model but an incorrect specified degree sequence. A systematic check whether different degree sequences (flat and skew) lead to different results over time would inform on such degree based effects. Second, we move further away from the IEA models and the independence assumption that they entail over time. Thus, social processes might be governing tie formation over time and the interdependence between their occurrences. A descriptive analysis of different network configurations might reveal such tendencies. For example, by doing a triad census over time, one can assess whether the effect of 'the friend of a friend is also a friend' is generating more closed triads over time given the actor covariates. Note that such assessments need to be performed on the original data, and not the aggregated multigraph representations.

## 8. Final remarks and future directions

We have presented and studied goodness of fit tests for random multigraph models defined by RSM and the closely related IEA models using the edge multiplicity sequence m of an observed multigraph with *n* vertices and *m* edges. Two particular kinds of IEA models are studied, both of which can be considered as approximations to the RSM models. These types of approximation have previously been shown to significantly facilitate the structural analysis of local and global properties of multigraphs [22,23]. Thus, it is of interest to know when the IEA models fit the data in such that statistics under these models can be used. To that end, the proposed goodness of fit tests can be used.

Note that we only have considered the consequences of replacing IEA models with RSM models, but have only tested IEA hypotheses. We include here some suggestions for future research on testing RSM hypotheses. A simple RSM( $d_{0}$) hypothesis has the same $Q_{0}$ as the IEAS( $d_{0}$) hypothesis, and $S_{0}$ and $A_{0}$ can not distinguish between these two hypotheses. Should the model be RSM( d), there is a dependency between edges when they are assigned to vertex pair sites, which could be used to distinguish between the two hypotheses. This requires a test not using $S_{0}$ or $A_{0}$, but a test using the full potential of m having as its critical region the set $\bar{M(d_{0})}$ consisting of all outcomes m that are not compatible with $d_{0}$. This test has zero probability of false rejection of RSM( $d_{0}$), and its power can be determined as the sum of the probabilities according to RSM( d) of the outcomes in the critical region. These RSM( d) probabilities and specifies outcomes of m compatible with a fixed degree sequence are presented in [23]. We leave this as suggestion for future research.

While the expected values of the Pearson statistic for simple hypotheses under the different models are derived, the exact expressions of both test statistic distributions are unknown. This implies that the numerical solutions which the presented tests depend upon are computationally expensive, thus restricting the tests to be practical on large scale multigraphs. A suggested extension to this work is to use Monte Carlo methods to study the distribution of test statistics and making the proposed tests applicable on larger multigraphs. This will also provide further insight to the asymptotic behavior of the test statistics and, in particular, how the dependence arising from edge assignments in the RSM model affects this behavior. Moreover, we have only considered the asymptotic analysis of power with respect to number of edges. Finding numerical and analytical ways in which this can be done based on number of vertices is yet another suggestion for future work.

## Supplementary Material

Supplemental Material

## Appendix. Derivations of expected values of test statistic E(S0)

### A.1. $E(S_{0})$ under ISA(p) model and $\mathrm{ISA}(p_{0})$ hypothesis

$$\begin{array}{rl} E(S_{0}) & =\sum_{i=1}^{n}L_{i}^{2}\left[1+(m-1)p_{i}^{2}\right]+\underset{i\neq j}{\sum \sum }\frac{L_{i}L_{j}}{2}\left[1+(m-1)2p_{i}p_{j}\right]-m\\ & =\sum_{i=1}^{n}L_{i}^{2}+(m-1)\sum_{i=1}^{n}(L_{i}p_{i}{)}^{2}+\sum_{i=1}^{n}\sum_{j=1}^{n}\frac{L_{i}L_{j}}{2}\\ & \;+(m-1)\sum_{i=1}^{n}\sum_{j=1}^{n}L_{i}L_{j}p_{i}p_{j}-\sum_{i=1}^{n}\frac{L_{i}^{2}}{2}-(m-1)\sum_{i=1}^{n}(L_{i}p_{i}{)}^{2}-m\\ & =\frac{\sum_{i=1}^{n}L_{i}^{2}+{\left(\sum_{i=1}^{n}L_{i}\right)}^{2}}{2}-m+(m-1){\left(\sum_{i=1}^{n}L_{i}p_{i}\right)}^{2} \end{array}$$

### A.2. $E(S_{0})$ under RSM(d) model and $\mathrm{RSM}(d_{0})$ or $\mathrm{IEAS}(d_{0})$ hypothesis

$$\begin{array}{rl} E(S_{0}) & =\underset{i\leq j}{\sum \sum }\frac{E(M_{ij}^{2})}{mQ_{0ij}}-m\\ & =\underset{i\leq j}{\sum \sum }\frac{\sigma_{ij}^{2}+\Delta_{ij}+m^{2}Q_{ij}^{2}}{mQ_{0ij}}-m\\ & =\underset{i\leq j}{\sum \sum }\frac{mQ_{ij}(1-Q_{ij})+\Delta_{ij}+m^{2}Q_{ij}^{2}}{mQ_{0ij}}-m \end{array}$$

For $Q=Q_{0}$, this simplifies to

$$\begin{array}{rl} E(S_{0}) & =r-1+\underset{i\leq j}{\sum \sum }\frac{\Delta_{ij}}{mQ_{ij}}=r-1+\underset{i\leq j}{\sum \sum }\frac{m(m-1)(Q_{ijij}-Q_{ij}^{2})}{mQ_{ij}}\\ & =r-1+(m-1)\left[\underset{i\leq j}{\sum \sum }\frac{Q_{ijij}}{Q_{ij}}-\underset{i\leq j}{\sum \sum }Q_{ij}\right]=r-m+(m-1)\left[\underset{i\leq j}{\sum \sum }\frac{Q_{ijij}}{Q_{ij}}\right]\\ & =r-m+(m-1)\left[\underset{i<j}{\sum \sum }\frac{2(d_{i}-1)(d_{j}-1)}{\left(2m-2)(2m-3\right)}+\sum_{i=1}^{n}\frac{\left(d_{i}-2)(d_{i}-3\right)}{\left(2m-2)(2m-3\right)}\right]\\ & =r-m+\frac{1}{2(2m-3)}\left[\underset{i\neq j}{\sum \sum }(d_{i}-1)(d_{j}-1)+\sum_{i=1}^{n}(d_{i}-2)(d_{i}-3)\right]\\ & =r-m\;+\frac{1}{2(2m-3)}\left[{\left(\sum_{i=1}^{n}(d_{i}-1)\right)}^{2}-\sum_{i=1}^{n}(d_{i}-1{)}^{2}+\sum_{i=1}^{n}d_{i}^{2}-5\sum_{i=1}^{n}d_{i}+6n\right]\\ & =r-m+\frac{1}{2(2m-3)}\left[4m^{2}+4mn+n^{2}-6m+5n\right]\\ & =\frac{\left(m-1)n(n-1\right)}{2m-3} \end{array}$$
