# Supplementary material for: Goodness of fit tests for random multigraph models
Source: J Appl Stat. 2022 Jul 21;50(15):3062–87. doi: 10.1080/02664763.2022.2099816 (PMC10631392; doi:10.1080/02664763.2022.2099816)
Supplement: Supplemental Material [file CJAS_A_2099816_SM1701.pdf]

Supplementary material to
"Goodness of fit tests for random multigraph models"

Termeh Shafie[a]

[a]GESIS – Leibniz Institute for the Social Sciences, Cologne, Germany

Contents

List of Figures 1

1 Simple IEA hypotheses against IEA models 3

2 Simple IEA hypotheses against RSM models 11

3 Composite IEA hypotheses against IEA models 18

4 Composite IEA hypotheses against RSM models 22

5 Data 25
5.1 Florentine family networks. 25
5.2 Friendship networks in a Dutch school class 26

List of Figures

1 Null distributions of S_0 and A_0, and the χ^2_9-distribution for some IEAS($\mathbf{d}$) models and IEAS($\mathbf{d}_0$) hypotheses with flat and skew $\mathbf{d}_0 = \mathbf{d}$ when m increases. 3
2 Non-null distributions of S_0 and A_0, and the χ^2_9-distribution for some IEAS($\mathbf{d}$) models and IEAS($\mathbf{d}_0$) hypotheses with flat and skew $\mathbf{d}_0 \neq \mathbf{d}$ when m increases. . 4
3 Power according to test statistics S_0 and A_0 when some simple ISA($\mathbf{p}_0$) hypotheses are tested against ISA($\mathbf{p}$) models for multigraphs with $n = 4$ and $m = 10$. The significance level for the asymptotic χ^2_9-distribution is 0.04. 5
4 Null distributions of S_0 and A_0, and the χ^2_9-distribution when some simple ISA($\mathbf{p}_0$) hypotheses are tested against ISA($\mathbf{p}$) models for multigraphs with $n = 4$ and $m = 10$. 6
5 Non-null distributions of S_0 and A_0, and the χ^2_9-distribution when some simple ISA($\mathbf{p}_0$) hypotheses are tested against ISA($\mathbf{p}$) models for multigraphs with $n = 4$ and $m = 10$. 7
6 Null distributions of S_0 and A_0, and the χ^2_9-distribution for some ISA($\mathbf{p}$) models and ISA($\mathbf{p}_0$) hypotheses with flat and skew $\mathbf{p}_0 = \mathbf{p}$ when m increases. 8
7 Non-null distributions of S_0 and A_0, and the χ^2_9-distribution for some ISA($\mathbf{p}$) models and ISA($\mathbf{p}_0$) hypotheses with flat and skew $\mathbf{p}_0 \neq \mathbf{p}$ when m increases. . . 9

Email: termeh.shafie@gesis.org

8 Preferred adjusted test statistics (top) and the degrees of freedom for preferred adjusted χ^2-distribution (bottom) for S_0 (left) and A_0 (right), when some simple ISA($\mathbf{p}_0$) hypotheses are tested against ISA($\mathbf{p}$) models for multigraphs with $n = 4$ and $m = 10$. 10
9 Power according to S_0 and A_0 when some simple IEAS($\mathbf{d}_0$) (top) and ISA($\mathbf{d}_0/2m$) (bottom) hypotheses are tested against RSM($\mathbf{d}$) models for multigraphs with $n = 4$ and $m = 30$. The significance level for the asymptotic χ^2_9-distribution is 0.04. Note that the degree sequences are ordered from skew to flat cases. 11
10 Non-null distributions of S_0 and A_0, and the χ^2_9-distribution when some simple IEAS($\mathbf{d}_0$) and ISA($\mathbf{d}_0/2m$) hypotheses are tested against RSM($\mathbf{d}$) models for multigraphs with $n = 4$ and $m = 30$. 12
11 Non-null distributions of S_0 and A_0 for some RSM($\mathbf{d}$) models and IEAS($\mathbf{d}_0$) hypotheses with flat $\mathbf{d}_0$ and different $\mathbf{d}$ when m increases. 13
12 Non-null distributions of S_0 and A_0 for some RSM($\mathbf{d}$) models and IEAS($\mathbf{d}_0$) hypotheses with skew $\mathbf{d}_0$ and different $\mathbf{d}$ when m increases. 14
13 Non-null distributions of S_0 and A_0 for some RSM($\mathbf{d}$) models and ISA($\mathbf{p}_0$) hypotheses with flat $\mathbf{p}_0$ and different $\mathbf{d}$ when m increases. 15
14 Non-null distributions of S_0 and A_0 for some RSM($\mathbf{d}$) models and ISA($\mathbf{p}_0$) hypotheses with skew $\mathbf{p}_0$ and different $\mathbf{d}$ when m increases. 16
15 Preferred adjusted test statistics (top) and the degrees of freedom for preferred adjusted χ^2-distribution (bottom) for S_0 (left) and A_0 (right), when some (a) simple IEAS($\mathbf{d}_0$) hypotheses, and (b) simple ISA($\mathbf{d}_0/2m$) hypotheses, are tested against RSM($\mathbf{d}$) models for multigraphs with $n = 4$ and $m = 30$. 17
16 Probabilities of false rejection (top) and power (bottom) according to test statistics $\hat{S}$ and $\hat{A}$ when some composite IEAS and ISA hypotheses are tested against (a) IEAS($\mathbf{d}$) models, and (b) ISA($\mathbf{p}$) models, for multigraphs with $n = 4$ and $m = 10$. The significance level for the asymptotic χ^2_6-distribution is 0.04. 18
17 Null and non-null distributions of $\hat{S}$, $\hat{A}$, and the χ^2_6-distribution when some composite IEAS and ISA hypotheses tested against IEAS($\mathbf{d}$) models for multigraphs with $n = 4$ and $m = 10$. 19
18 Null and non-null distributions of $\hat{S}$ and $\hat{A}$ for some IEAS($\mathbf{d}$) models with flat and skew $\mathbf{d}$, and composite IEAS and ISA hypotheses when m increases. 20
19 Preferred adjusted test statistics (top) and the degrees of freedom for preferred adjusted χ^2-distribution (bottom) for $\hat{S}$ (left) and $\hat{A}$ (right), when some composite IEAS hypotheses are tested against IEAS($\mathbf{d}$) models for multigraphs with $n = 4$ and $m = 10$. 21
20 Preferred adjusted test statistics (top) and the degrees of freedom for preferred adjusted χ^2-distribution (bottom) for $\hat{S}$ (left) and $\hat{A}$ (right), when some composite ISA hypotheses are tested against ISA($\mathbf{p}$) models for multigraphs with $n = 4$ and $m = 10$. 21
21 Power according to test statistics $\hat{S}$ and $\hat{A}$ when some composite IEAS and ISA hypotheses are tested against RSM($\mathbf{d}$) models for multigraphs with $n = 4$ and $m = 30$. The significance level for the asymptotic χ^2_6-distribution is 0.04. 22
22 Non-null distributions of $\hat{S}$, $\hat{A}$, and the χ^2_6-distribution when some composite IEAS and ISA hypotheses tested against RSM($\mathbf{d}$) models for multigraphs with $n = 4$ and $m = 30$. 23
23 Non-null distributions of $\hat{S}$ and $\hat{A}$ for some RSM($\mathbf{d}$) models with flat and skew $\mathbf{d}$, and composite IEAS and ISA hypotheses when m increases. 24

1. Simple IEA hypotheses against IEA models

Figures with respect to section 5.1 in main article where simple ISA multigraph hypotheses are tested against ISA models.

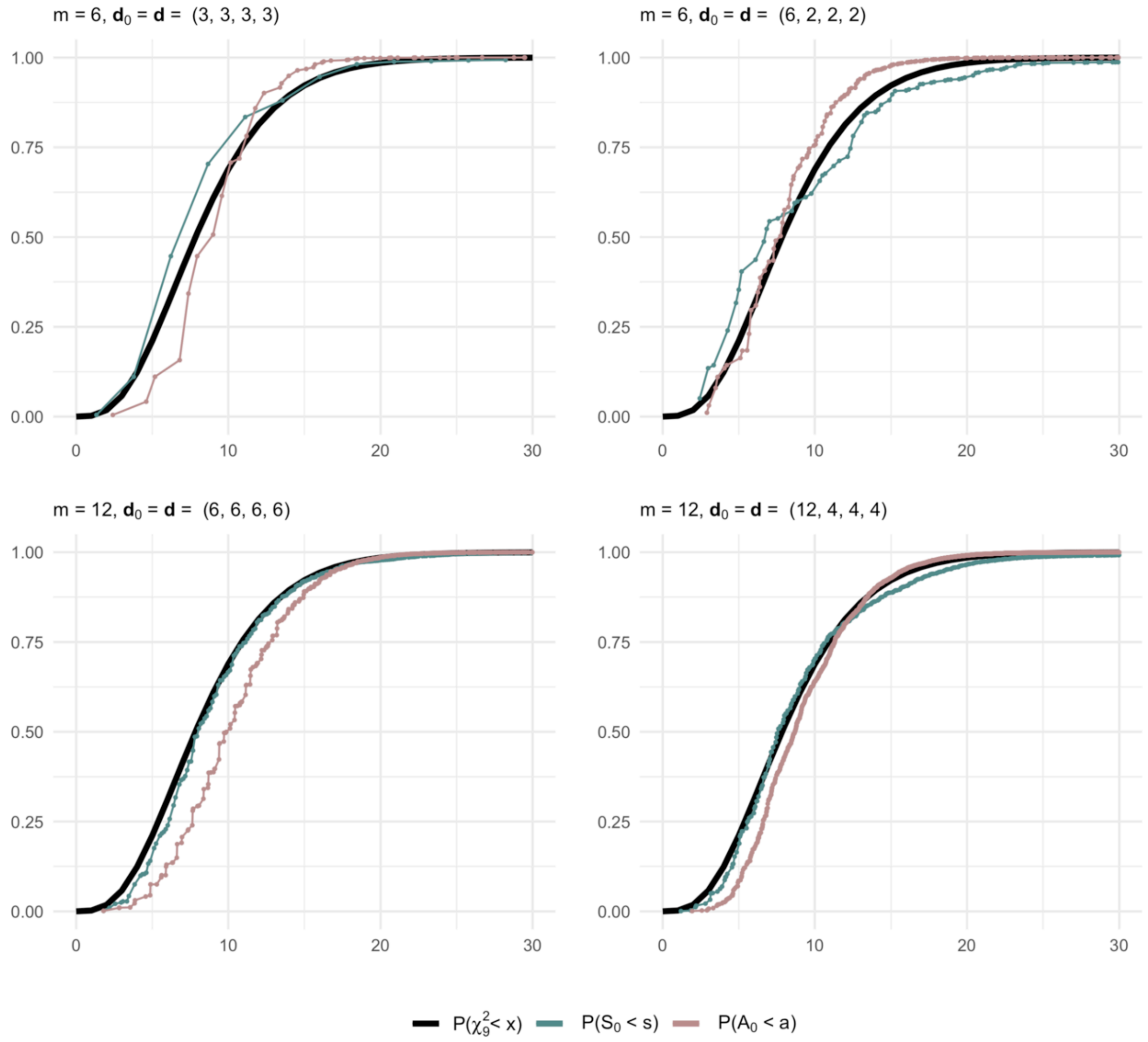

Figure 1. Null distributions of S_0 and A_0, and the χ^2_9-distribution for some IEAS($\mathbf{d}$) models and IEAS($\mathbf{d}_0$) hypotheses with flat and skew $\mathbf{d}_0 = \mathbf{d}$ when m increases.

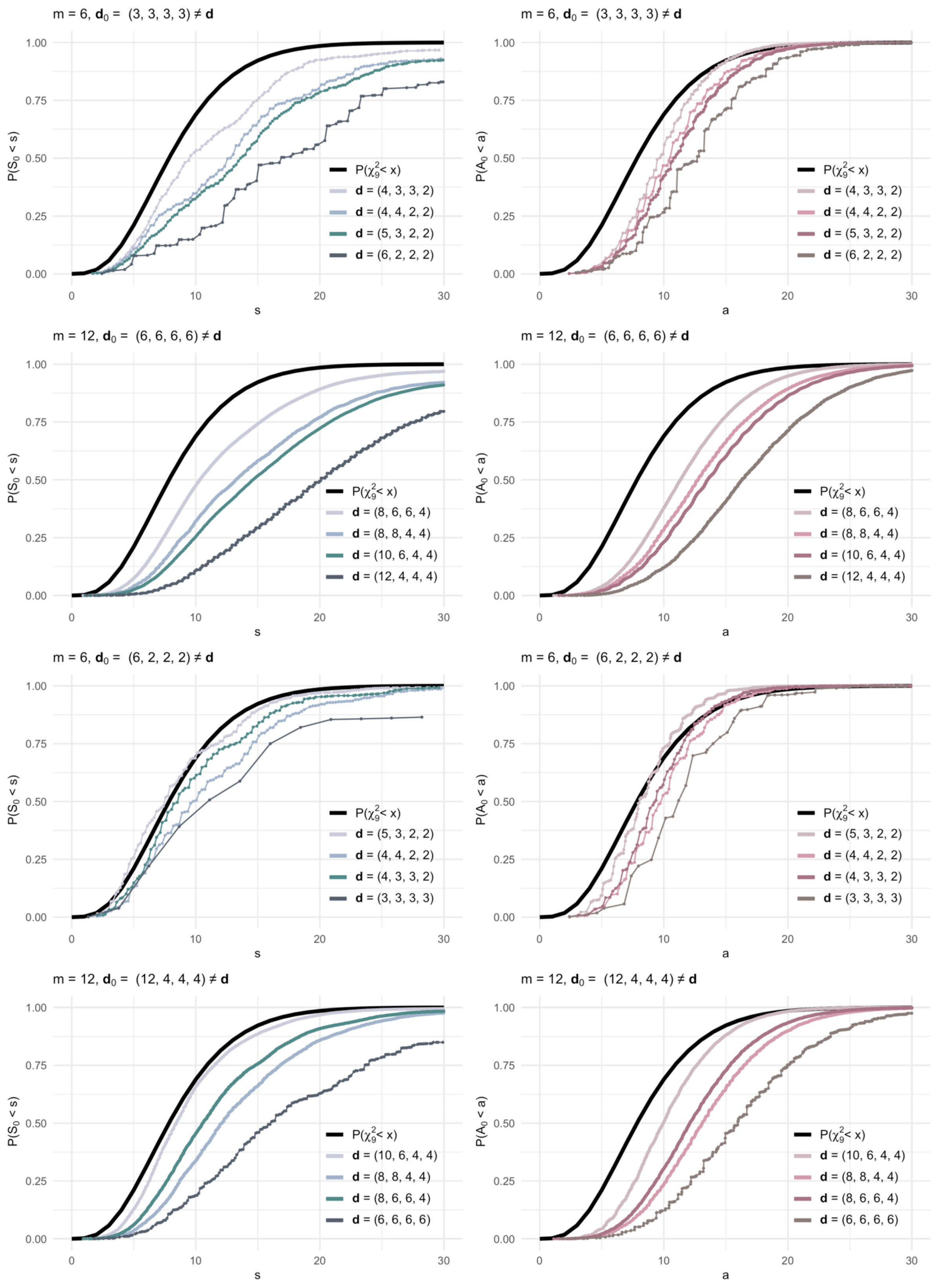

Figure 2. Non-null distributions of S_0 and A_0, and the χ^2_9-distribution for some IEAS($\mathbf{d}$) models and IEAS($\mathbf{d}_0$) hypotheses with flat and skew $\mathbf{d}_0 \neq \mathbf{d}$ when m increases.

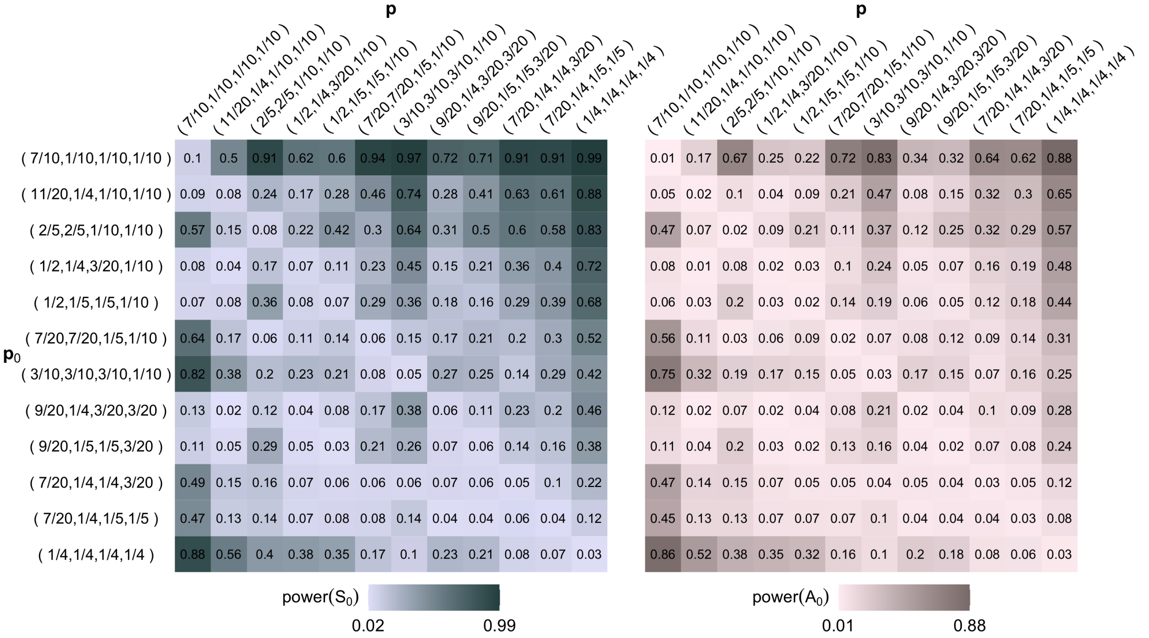

Figure 3. Power according to test statistics S_0 and A_0 when some simple ISA($\mathbf{p}_0$) hypotheses are tested against ISA($\mathbf{p}$) models for multigraphs with $n = 4$ and $m = 10$. The significance level for the asymptotic χ^2_9-distribution is 0.04.

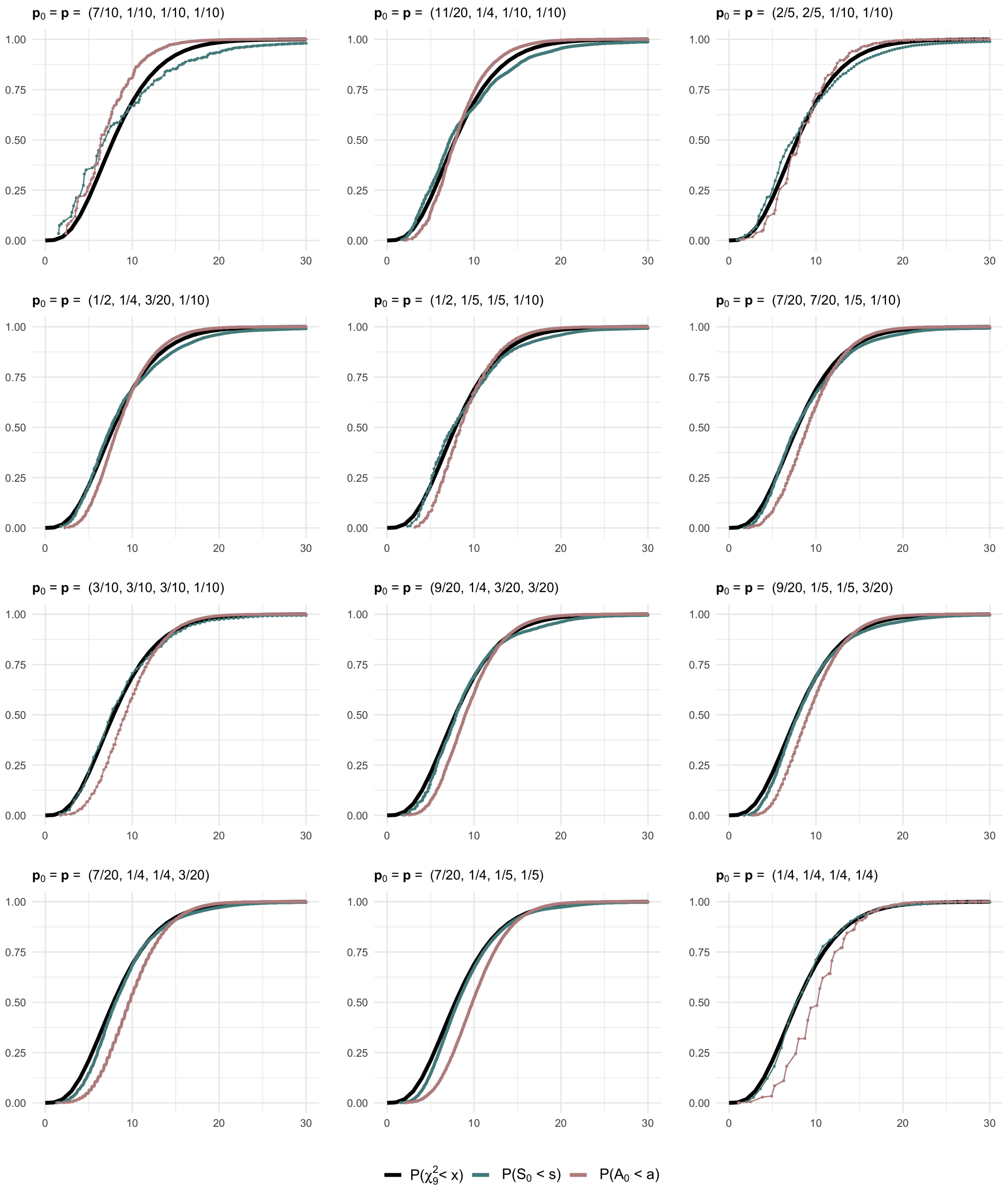

Figure 4. Null distributions of S_0 and A_0, and the χ^2_9-distribution when some simple ISA($\mathbf{p}_0$) hypotheses are tested against ISA($\mathbf{p}$) models for multigraphs with $n = 4$ and $m = 10$.

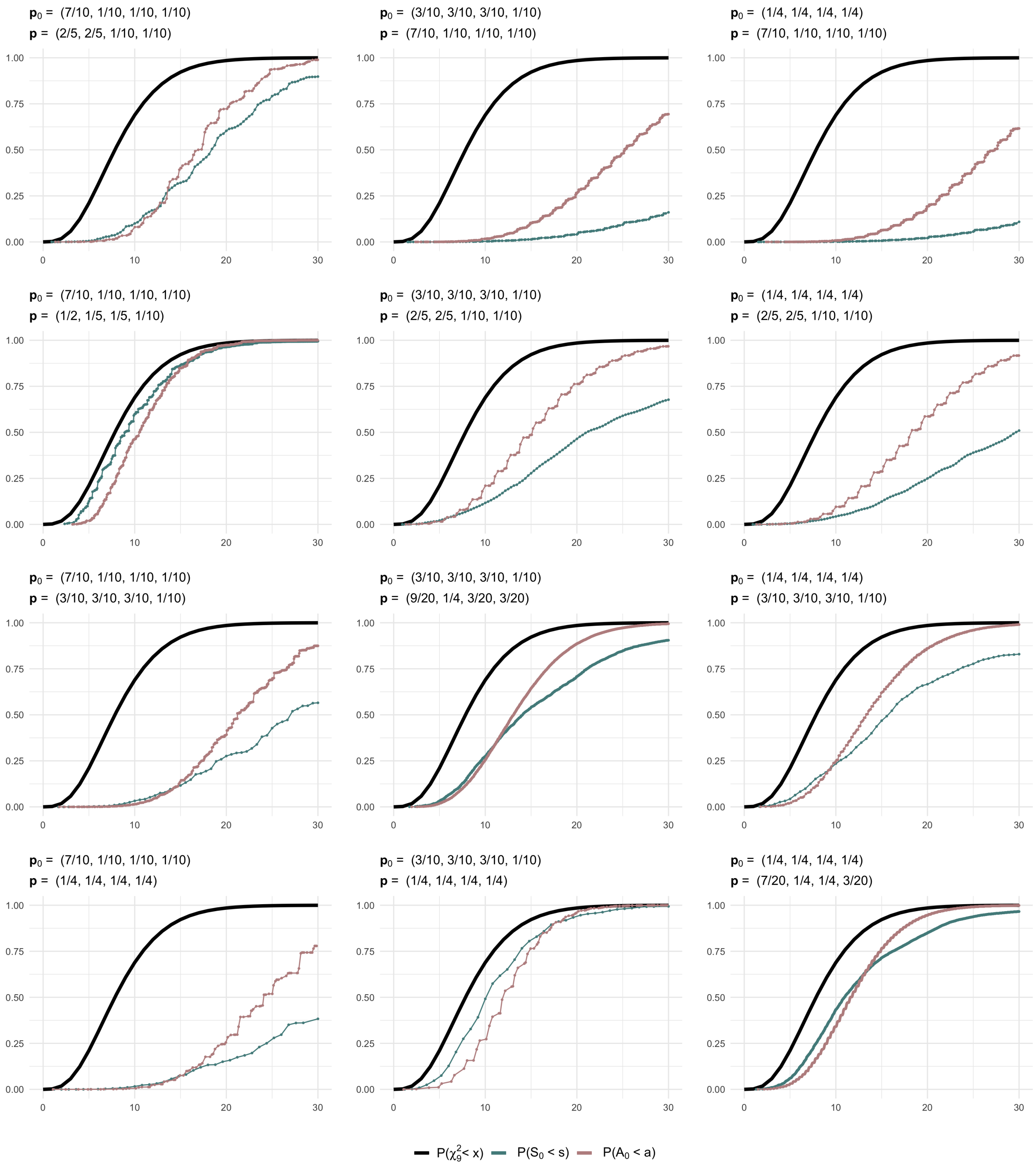

Figure 5. Non-null distributions of S_0 and A_0, and the χ^2_9-distribution when some simple ISA($\mathbf{p}_0$) hypotheses are tested against ISA($\mathbf{p}$) models for multigraphs with $n = 4$ and $m = 10$.

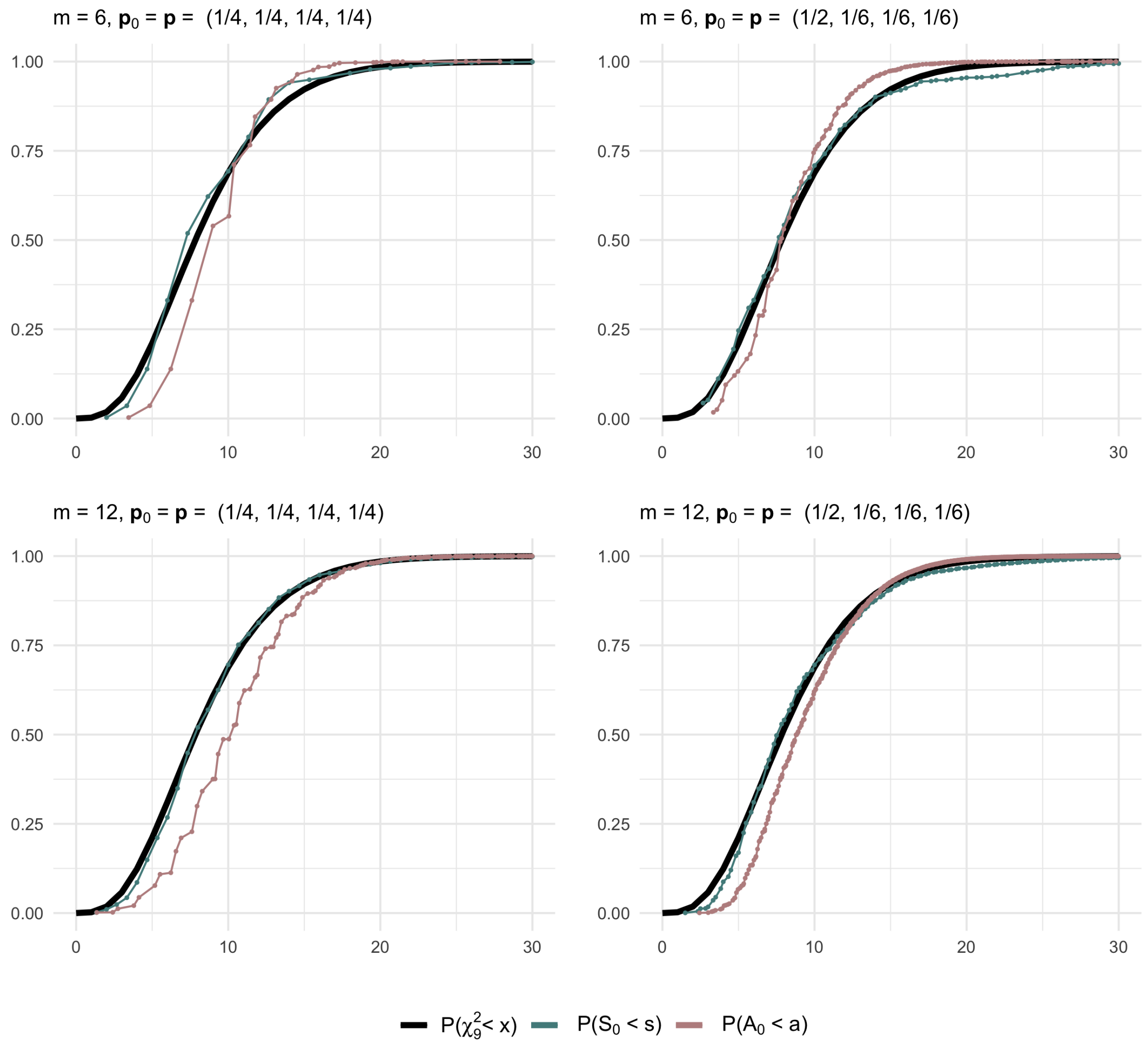

Figure 6. Null distributions of S_0 and A_0, and the χ^2_9-distribution for some ISA($\mathbf{p}$) models and ISA($\mathbf{p}_0$) hypotheses with flat and skew $\mathbf{p}_0 = \mathbf{p}$ when m increases.

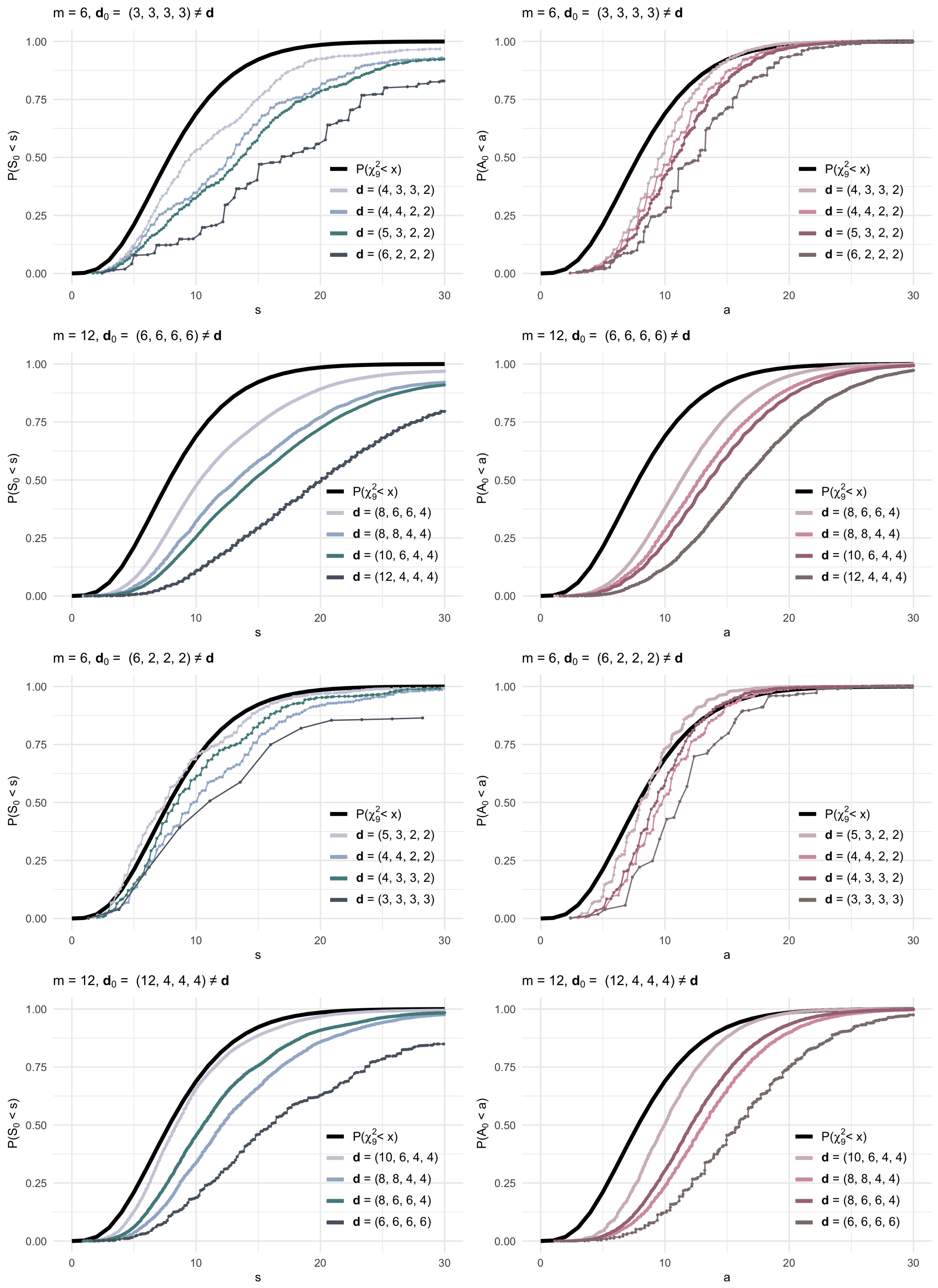

Figure 7. Non-null distributions of S_0 and A_0, and the χ^2_9-distribution for some ISA($\mathbf{p}$) models and ISA($\mathbf{p}_0$) hypotheses with flat and skew $\mathbf{p}_0 \neq \mathbf{p}$ when m increases.

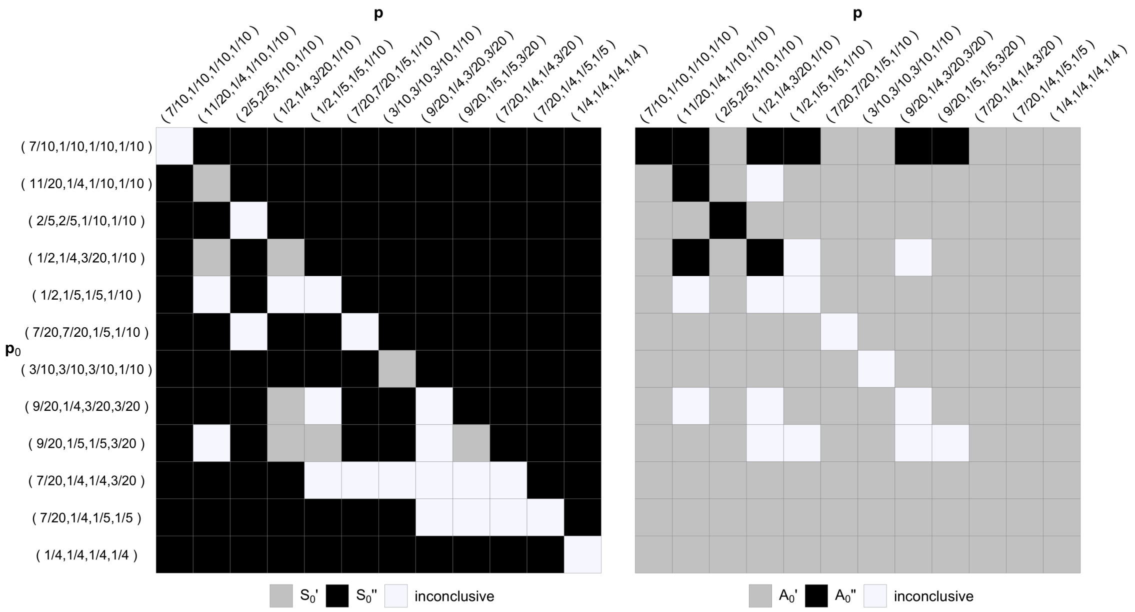

Degrees of freedom for S_0 (rows: $\mathbf{p}_0$; columns: $\mathbf{p}$):

$\mathbf{p}_0$ \ $\mathbf{p}$	(7/10,1/10,1/10,1/10)	(11/20,1/4,1/10,1/10)	(2/5,2/5,1/10,1/10)	(1/2,1/4,3/20,1/10)	(1/2,1/5,1/5,1/10)	(7/20,7/20,1/5,1/10)	(3/10,3/10,3/10,1/10)	(9/20,1/4,3/20,3/20)	(9/20,1/5,1/5,3/20)	(7/20,1/4,1/4,3/20)	(7/20,1/4,1/5,1/5)	(1/4,1/4,1/4,1/4)
(7/10,1/10,1/10,1/10)	9	9	9	9	9	9	9	9	9	9	9	9
(11/20,1/4,1/10,1/10)	9	8	9	9	9	9	9	9	9	9	9	9
(2/5,2/5,1/10,1/10)	9	9	9	9	9	9	9	9	9	9	9	9
(1/2,1/4,3/20,1/10)	9	8	9	8	9	9	9	9	9	9	9	9
(1/2,1/5,1/5,1/10)	9	9	9	9	9	9	9	9	9	9	9	9
(7/20,7/20,1/5,1/10)	9	9	9	9	9	9	9	9	9	9	9	9
(3/10,3/10,3/10,1/10)	9	9	9	9	9	9	8	9	9	9	9	9
(9/20,1/4,3/20,3/20)	9	9	9	8	9	9	9	9	9	9	9	9
(9/20,1/5,1/5,3/20)	9	9	9	8	8	9	9	9	8	9	9	9
(7/20,1/4,1/4,3/20)	9	9	9	9	9	9	9	9	9	9	9	9
(7/20,1/4,1/5,1/5)	9	9	9	9	9	9	9	9	9	9	9	9
(1/4,1/4,1/4,1/4)	9	9	9	9	9	9	9	9	9	9	9	9

Degrees of freedom for A_0 (rows: $\mathbf{p}_0$; columns: $\mathbf{p}$):

$\mathbf{p}_0$ \ $\mathbf{p}$	(7/10,1/10,1/10,1/10)	(11/20,1/4,1/10,1/10)	(2/5,2/5,1/10,1/10)	(1/2,1/4,3/20,1/10)	(1/2,1/5,1/5,1/10)	(7/20,7/20,1/5,1/10)	(3/10,3/10,3/10,1/10)	(9/20,1/4,3/20,3/20)	(9/20,1/5,1/5,3/20)	(7/20,1/4,1/4,3/20)	(7/20,1/4,1/5,1/5)	(1/4,1/4,1/4,1/4)
(7/10,1/10,1/10,1/10)	9	9	9	9	9	9	25	9	9	9	20	27
(11/20,1/4,1/10,1/10)	10	9	11	9	10	13	17	10	12	15	14	20
(2/5,2/5,1/10,1/10)	17	10	9	11	13	11	9	11	14	15	14	19
(1/2,1/4,3/20,1/10)	11	9	10	9	9	11	14	9	10	12	13	17
(1/2,1/5,1/5,1/10)	11	9	13	9	9	12	13	10	10	11	12	17
(7/20,7/20,1/5,1/10)	18	12	10	10	11	9	10	11	12	11	12	15
(3/10,3/10,3/10,1/10)	22	15	13	13	12	10	9	13	12	10	12	14
(9/20,1/4,3/20,3/20)	12	9	11	9	10	11	13	9	10	11	11	14
(9/20,1/5,1/5,3/20)	12	10	13	9	9	12	12	9	9	10	11	14
(7/20,1/4,1/4,3/20)	17	13	13	11	10	10	10	10	10	10	10	12
(7/20,1/4,1/5,1/5)	17	12	12	11	11	11	12	10	10	10	10	11
(1/4,1/4,1/4,1/4)	24	18	16	16	15	13	12	13	13	11	11	10

Figure 8. Preferred adjusted test statistics (top) and the degrees of freedom for preferred adjusted χ^2-distribution (bottom) for S_0 (left) and A_0 (right), when some simple ISA($\mathbf{p}_0$) hypotheses are tested against ISA($\mathbf{p}$) models for multigraphs with $n = 4$ and $m = 10$.

2. Simple IEA hypotheses against RSM models

Figures with respect to section 5.2 in main article where simple IEAS and ISA multigraph hypotheses are tested against RSM models.

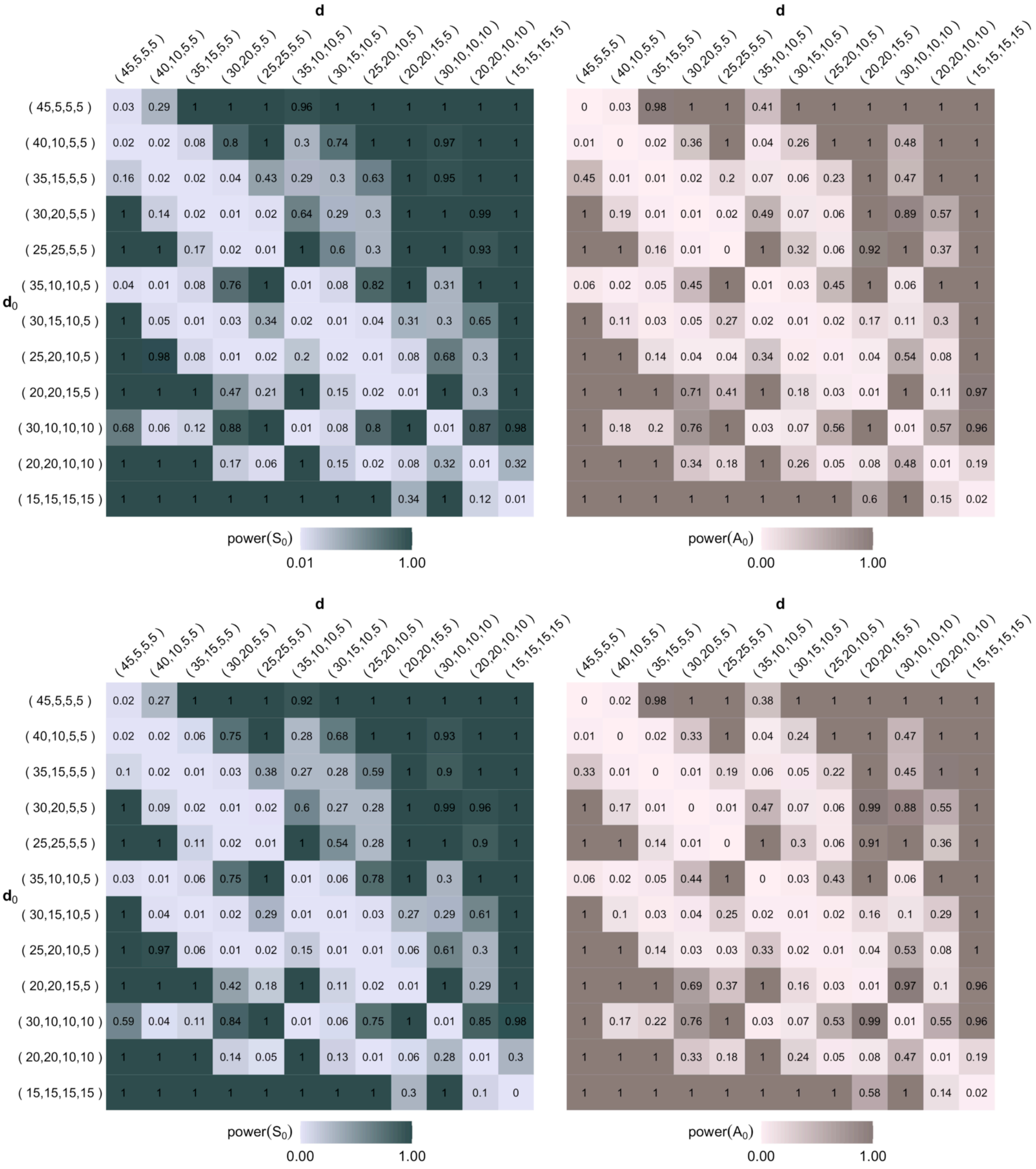

Figure 9. Power according to S_0 and A_0 when some simple IEAS($\mathbf{d}_0$) (top) and ISA($\mathbf{d}_0/2m$) (bottom) hypotheses are tested against RSM($\mathbf{d}$) models for multigraphs with $n = 4$ and $m = 30$. The significance level for the asymptotic χ^2_9-distribution is 0.04. Note that the degree sequences are ordered from skew to flat cases.

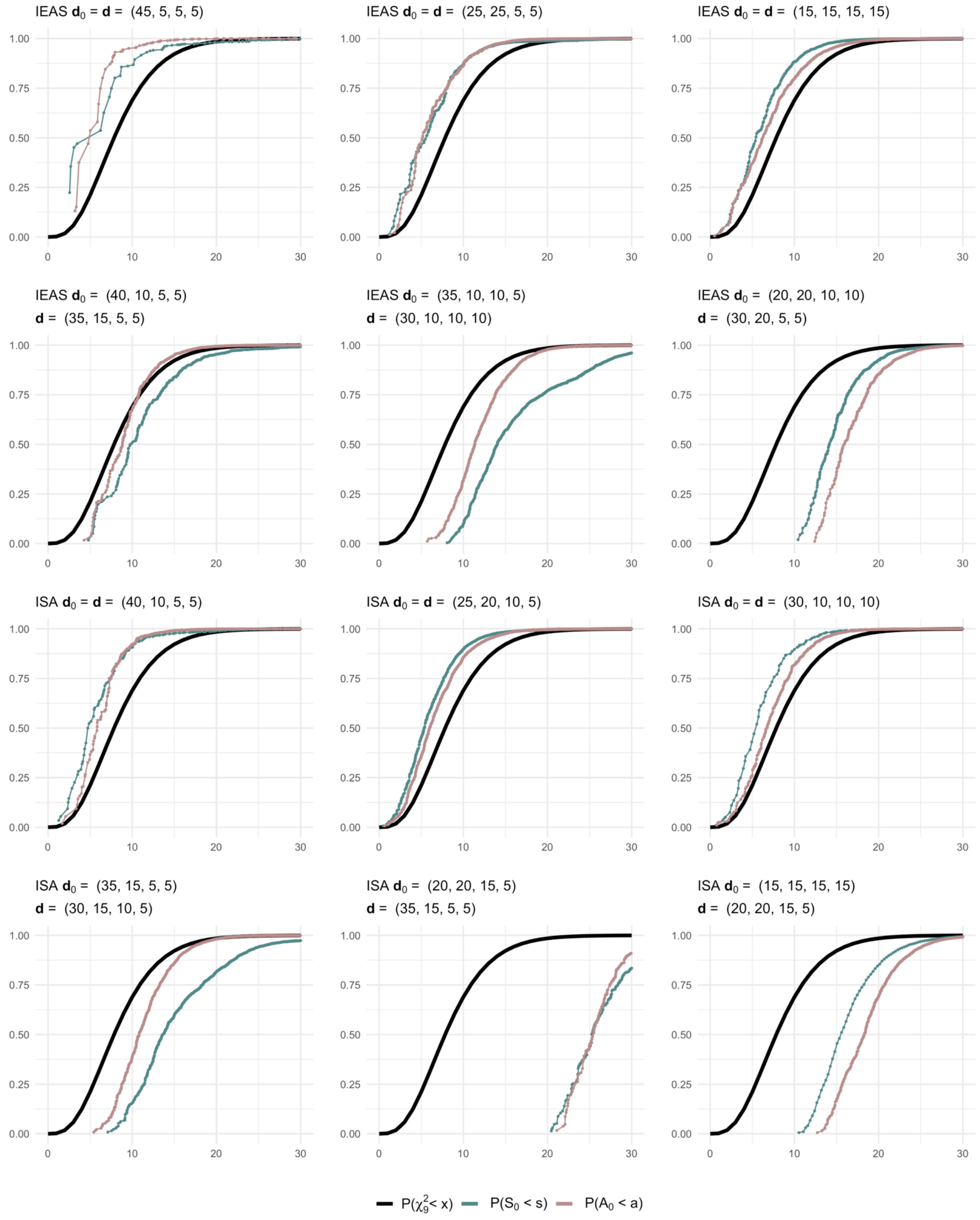

Figure 10. Non-null distributions of S_0 and A_0, and the χ^2_9-distribution when some simple IEAS($\mathbf{d}_0$) and ISA($\mathbf{d}_0/2m$) hypotheses are tested against RSM($\mathbf{d}$) models for multigraphs with $n = 4$ and $m = 30$.

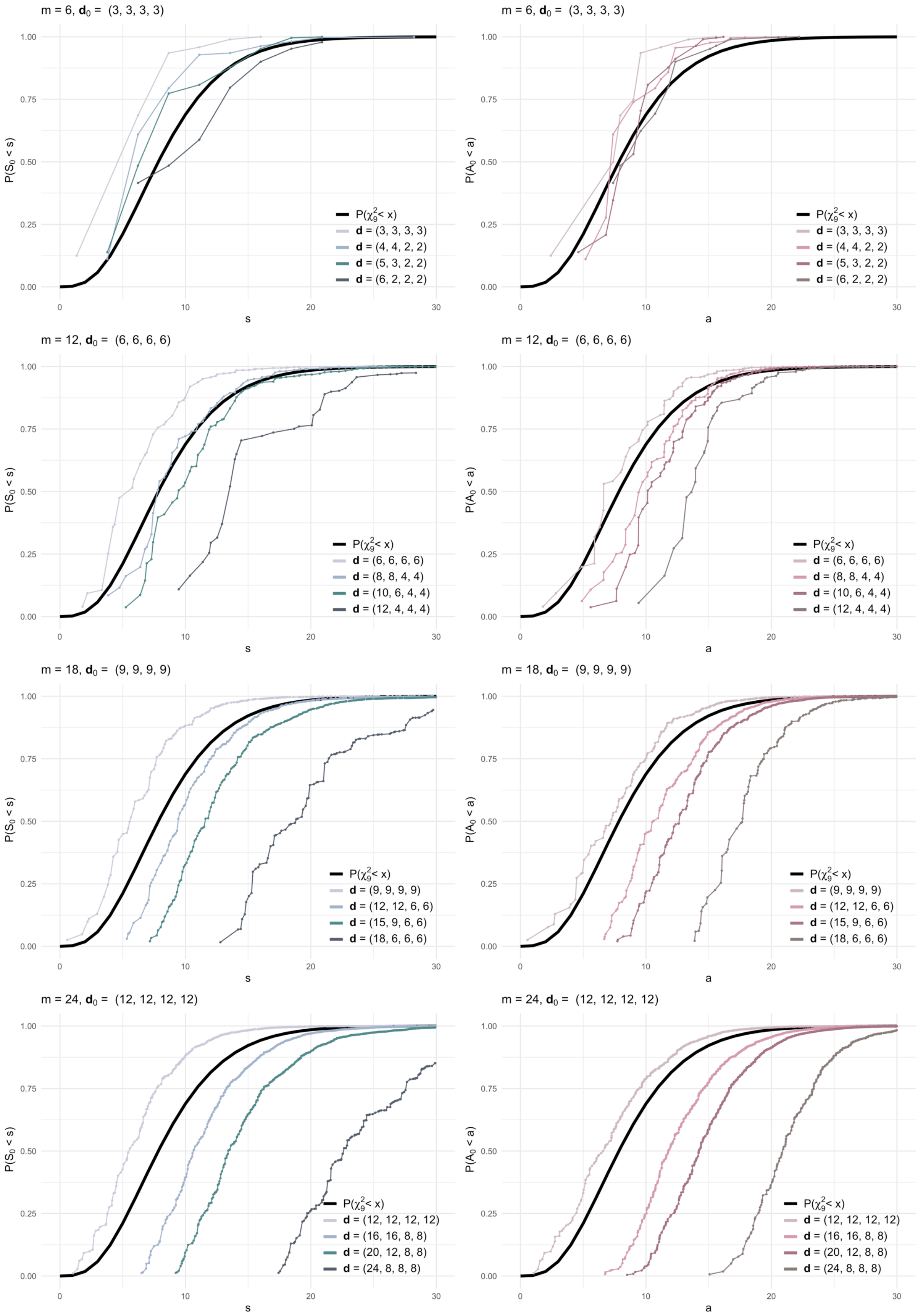

Figure 11. Non-null distributions of S_0 and A_0 for some RSM($\mathbf{d}$) models and IEAS($\mathbf{d}_0$) hypotheses with flat $\mathbf{d}_0$ and different $\mathbf{d}$ when m increases.

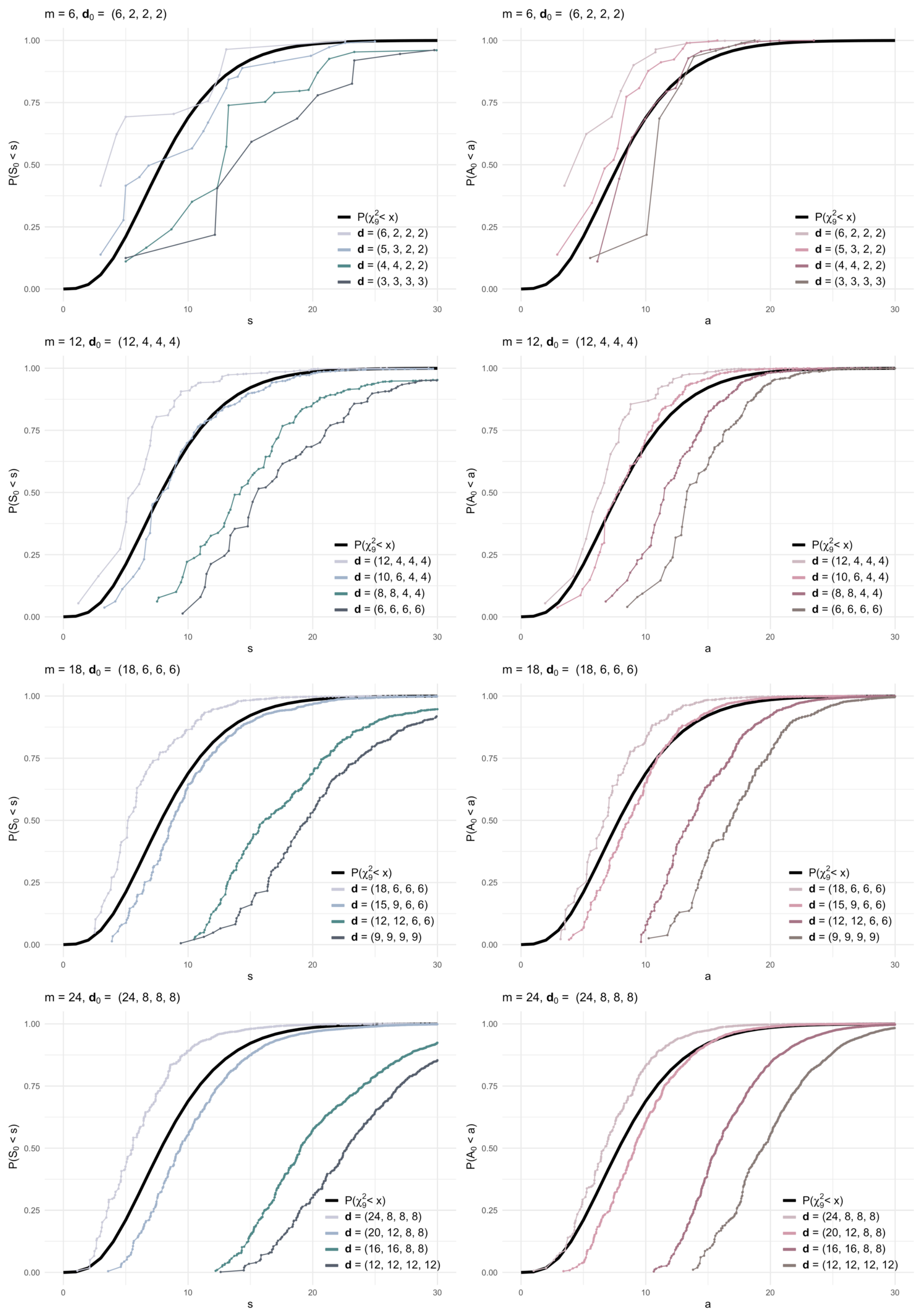

Figure 12. Non-null distributions of S_0 and A_0 for some RSM($\mathbf{d}$) models and IEAS($\mathbf{d}_0$) hypotheses with skew $\mathbf{d}_0$ and different $\mathbf{d}$ when m increases.

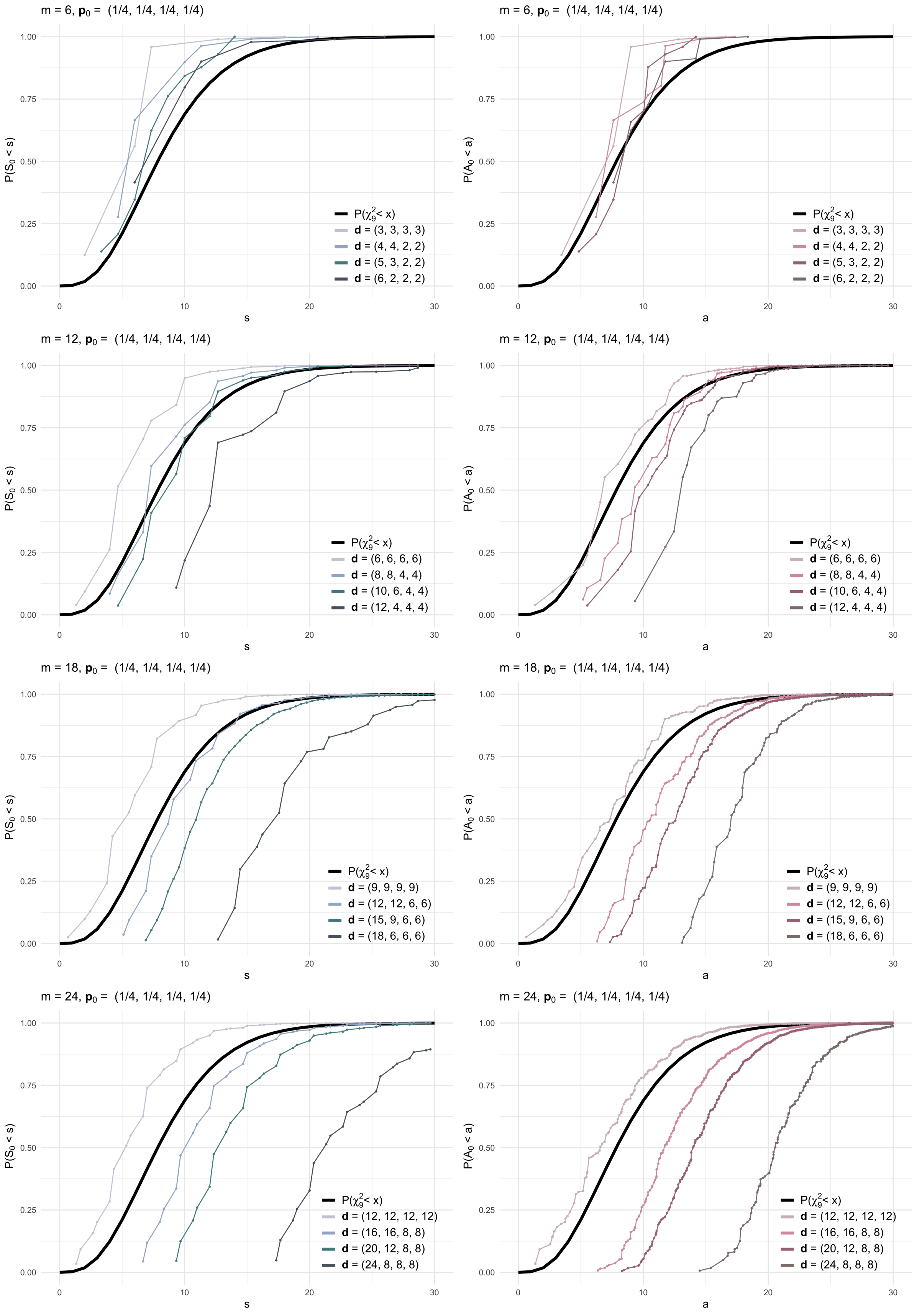

Figure 13. Non-null distributions of S_0 and A_0 for some RSM($\mathbf{d}$) models and ISA($\mathbf{p}_0$) hypotheses with flat $\mathbf{p}_0$ and different $\mathbf{d}$ when m increases.

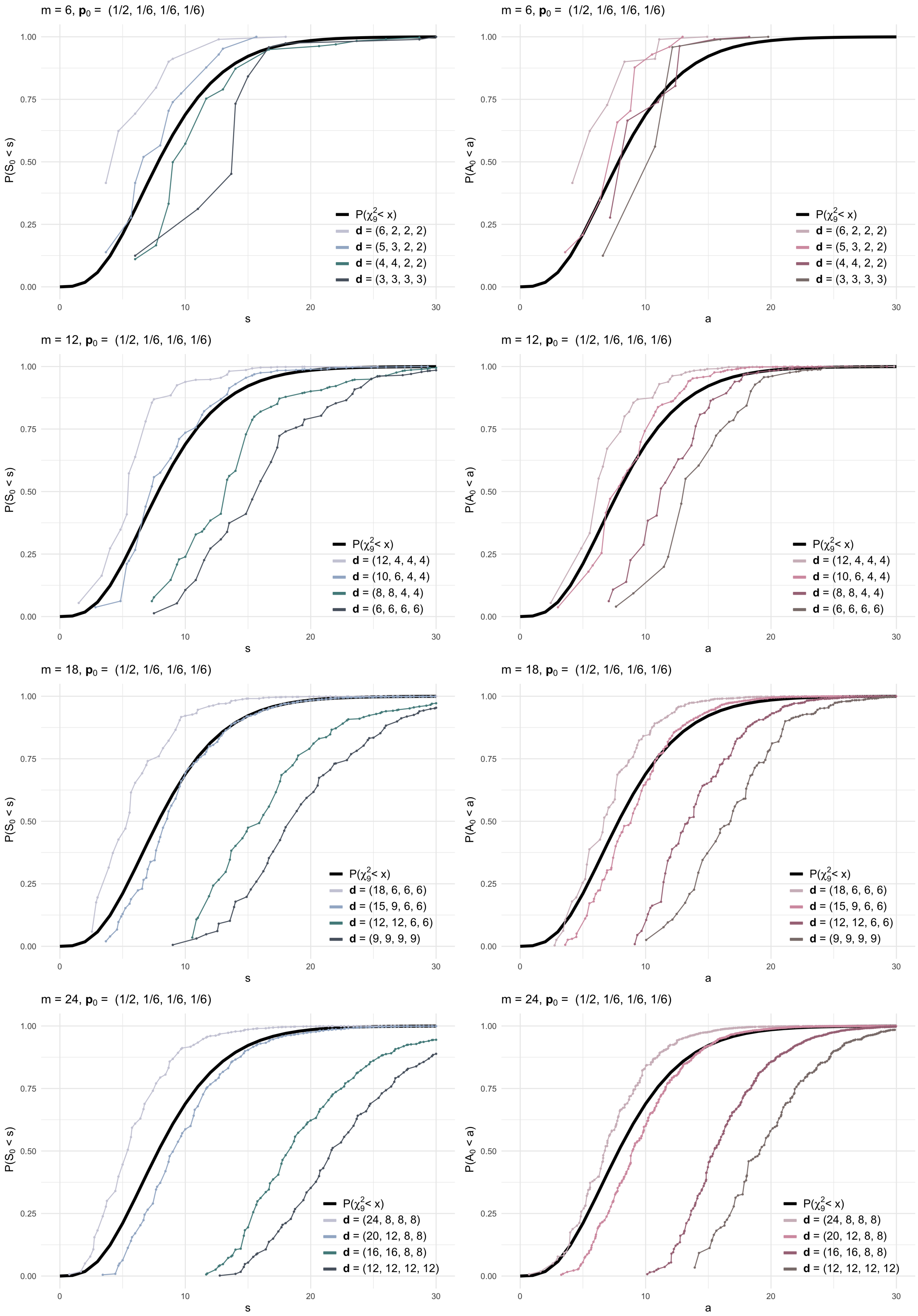

Figure 14. Non-null distributions of S_0 and A_0 for some RSM($\mathbf{d}$) models and ISA($\mathbf{p}_0$) hypotheses with skew $\mathbf{p}_0$ and different $\mathbf{d}$ when m increases.

(a)

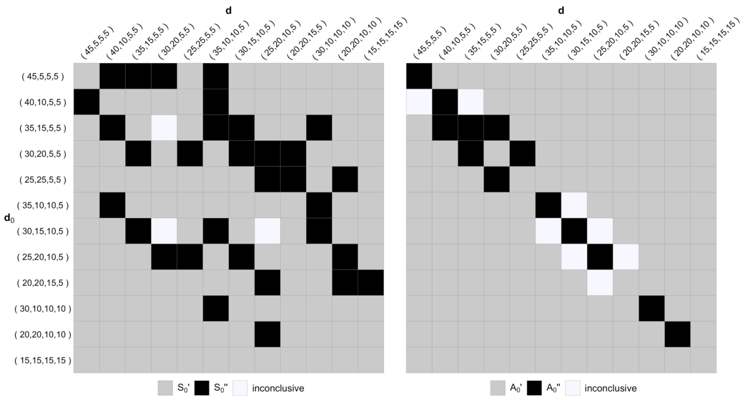

S_0 (left), (a):

$\mathbf{d}_0$ \ $\mathbf{d}$	(45,5,5,5)	(40,10,5,5)	(35,15,5,5)	(30,20,5,5)	(25,25,5,5)	(35,10,10,5)	(30,15,10,5)	(25,20,10,5)	(20,20,15,5)	(30,10,10,10)	(20,20,10,10)	(15,15,15,15)
(45,5,5,5)	6	9	9	9	9	9	9	9	9	9	9	9
(40,10,5,5)	9	6	10	23	45	9	9	38	9	9	56	9
(35,15,5,5)	14	9	6	9	17	9	9	9	9	9	9	9
(30,20,5,5)	26	14	9	6	9	21	9	9	9	9	9	9
(25,25,5,5)	45	26	14	8	6	30	20	9	9	37	9	9
(35,10,10,5)	11	9	11	22	43	6	10	23	32	9	39	9
(30,15,10,5)	22	11	9	9	16	9	6	9	16	9	21	9
(25,20,10,5)	40	22	12	9	9	15	9	6	10	21	9	9
(20,20,15,5)	74	44	27	17	15	25	13	9	6	28	9	9
(30,10,10,10)	19	12	13	23	42	9	11	22	29	6	23	27
(20,20,10,10)	70	41	23	14	12	26	14	9	11	16	6	16
(15,15,15,15)	141	90	59	43	38	53	33	24	16	29	12	6

A_0 (right), (a):

$\mathbf{d}_0$ \ $\mathbf{d}$	(45,5,5,5)	(40,10,5,5)	(35,15,5,5)	(30,20,5,5)	(25,25,5,5)	(35,10,10,5)	(30,15,10,5)	(25,20,10,5)	(20,20,15,5)	(30,10,10,10)	(20,20,10,10)	(15,15,15,15)
(45,5,5,5)	9	10	21	38	58	17	29	47	64	24	58	74
(40,10,5,5)	9	9	9	16	28	11	15	25	40	17	35	56
(35,15,5,5)	17	9	9	9	14	12	11	15	29	17	24	48
(30,20,5,5)	28	15	9	6	9	17	12	11	23	21	18	44
(25,25,5,5)	42	25	14	9	6	26	16	11	22	27	17	43
(35,10,10,5)	14	10	11	17	28	9	9	17	24	11	26	39
(30,15,10,5)	24	14	10	10	15	9	9	9	14	13	16	31
(25,20,10,5)	38	23	14	10	10	16	9	6	9	18	11	28
(20,20,15,5)	54	37	26	19	17	24	14	9	6	23	12	23
(30,10,10,10)	21	15	15	20	29	10	12	18	23	9	18	22
(20,20,10,10)	51	34	23	16	14	25	15	10	11	17	6	14
(15,15,15,15)	72	55	44	37	35	39	29	24	18	24	13	6

(b)

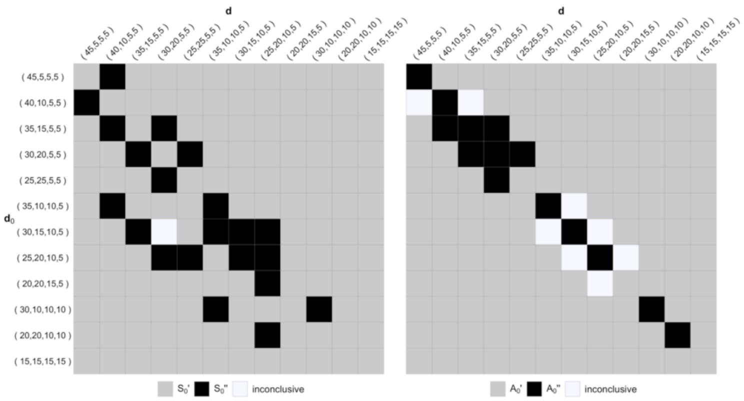

S_0 (left), (b):

$\mathbf{d}_0$ \ $\mathbf{d}$	(45,5,5,5)	(40,10,5,5)	(35,15,5,5)	(30,20,5,5)	(25,25,5,5)	(35,10,10,5)	(30,15,10,5)	(25,20,10,5)	(20,20,15,5)	(30,10,10,10)	(20,20,10,10)	(15,15,15,15)
(45,5,5,5)	5	9	9	9	9	9	9	9	9	9	9	9
(40,10,5,5)	9	5	10	21	42	9	21	36	9	9	54	9
(35,15,5,5)	14	9	5	9	16	15	9	20	9	9	34	9
(30,20,5,5)	25	13	9	5	9	19	15	9	9	29	27	9
(25,25,5,5)	44	25	13	9	5	28	19	15	9	35	9	9
(35,10,10,5)	11	9	10	21	40	5	10	22	31	15	37	63
(30,15,10,5)	21	11	9	9	16	9	5	9	15	15	20	9
(25,20,10,5)	39	22	12	9	9	14	9	5	10	20	15	9
(20,20,15,5)	71	43	26	17	14	24	13	9	5	26	15	9
(30,10,10,10)	18	12	13	22	40	9	10	21	28	6	22	26
(20,20,10,10)	67	39	23	14	11	25	13	9	11	16	6	15
(15,15,15,15)	133	85	56	41	37	51	32	24	16	27	12	6

A_0 (right), (b):

$\mathbf{d}_0$ \ $\mathbf{d}$	(45,5,5,5)	(40,10,5,5)	(35,15,5,5)	(30,20,5,5)	(25,25,5,5)	(35,10,10,5)	(30,15,10,5)	(25,20,10,5)	(20,20,15,5)	(30,10,10,10)	(20,20,10,10)	(15,15,15,15)
(45,5,5,5)	9	10	21	37	57	16	29	46	62	24	57	72
(40,10,5,5)	9	9	9	16	28	11	15	24	39	17	34	55
(35,15,5,5)	17	9	9	9	14	12	11	15	28	17	23	47
(30,20,5,5)	28	15	9	6	9	17	11	11	23	21	18	43
(25,25,5,5)	42	25	14	9	6	25	16	11	21	27	16	42
(35,10,10,5)	14	10	11	17	28	9	9	17	24	11	26	38
(30,15,10,5)	24	14	10	10	15	9	9	9	14	12	16	31
(25,20,10,5)	37	23	14	10	10	16	9	9	9	18	11	28
(20,20,15,5)	53	36	25	19	17	24	14	9	6	23	12	22
(30,10,10,10)	21	15	15	19	28	10	11	18	23	9	18	22
(20,20,10,10)	50	33	22	16	14	25	15	10	11	17	6	13
(15,15,15,15)	71	54	43	37	35	38	29	24	18	24	13	6

Figure 15. Preferred adjusted test statistics (top) and the degrees of freedom for preferred adjusted χ^2-distribution (bottom) for S_0 (left) and A_0 (right), when some (a) simple IEAS($\mathbf{d}_0$) hypotheses, and (b) simple ISA($\mathbf{d}_0/2m$) hypotheses, are tested against RSM($\mathbf{d}$) models for multigraphs with $n = 4$ and $m = 30$.

3. Composite IEA hypotheses against IEA models

Figures with respect to section 5.3 in main article where composite IEA multigraph hypotheses are tested against IEA models.

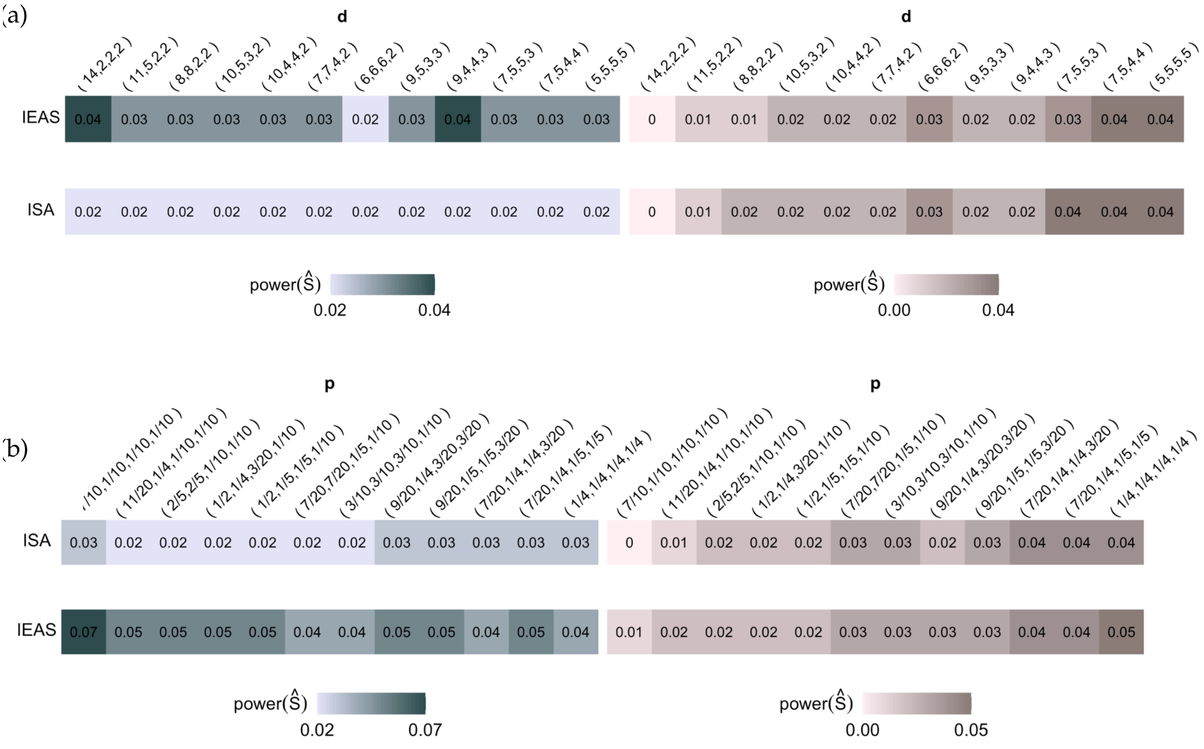

Figure 16. Probabilities of false rejection (top) and power (bottom) according to test statistics $\hat{S}$ and $\hat{A}$ when some composite IEAS and ISA hypotheses are tested against (a) IEAS($\mathbf{d}$) models, and (b) ISA($\mathbf{p}$) models, for multigraphs with $n = 4$ and $m = 10$. The significance level for the asymptotic χ^2_6-distribution is 0.04.

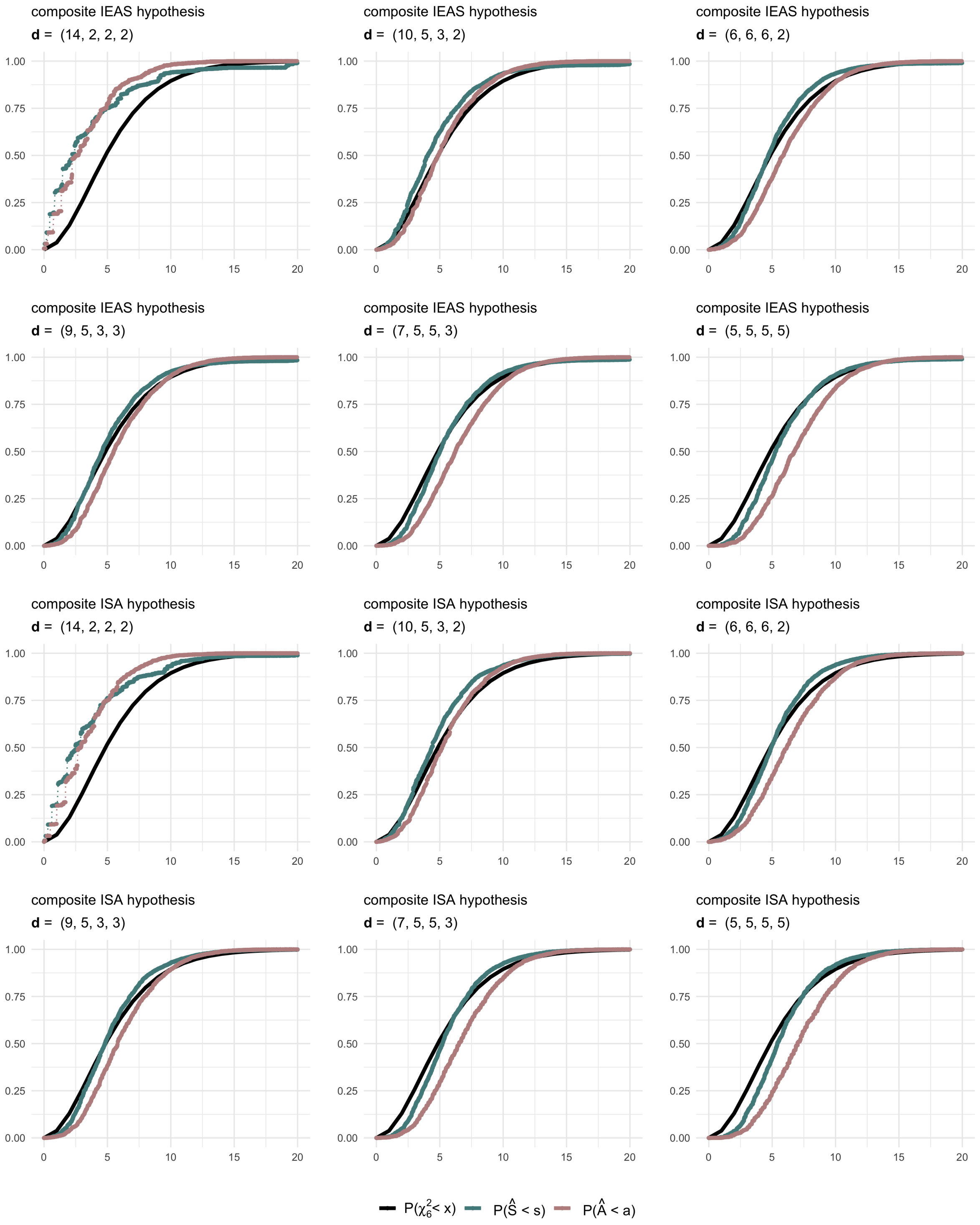

Figure 17. Null and non-null distributions of $\hat{S}$, $\hat{A}$, and the χ^2_6-distribution when some composite IEAS and ISA hypotheses tested against IEAS($\mathbf{d}$) models for multigraphs with $n = 4$ and $m = 10$.

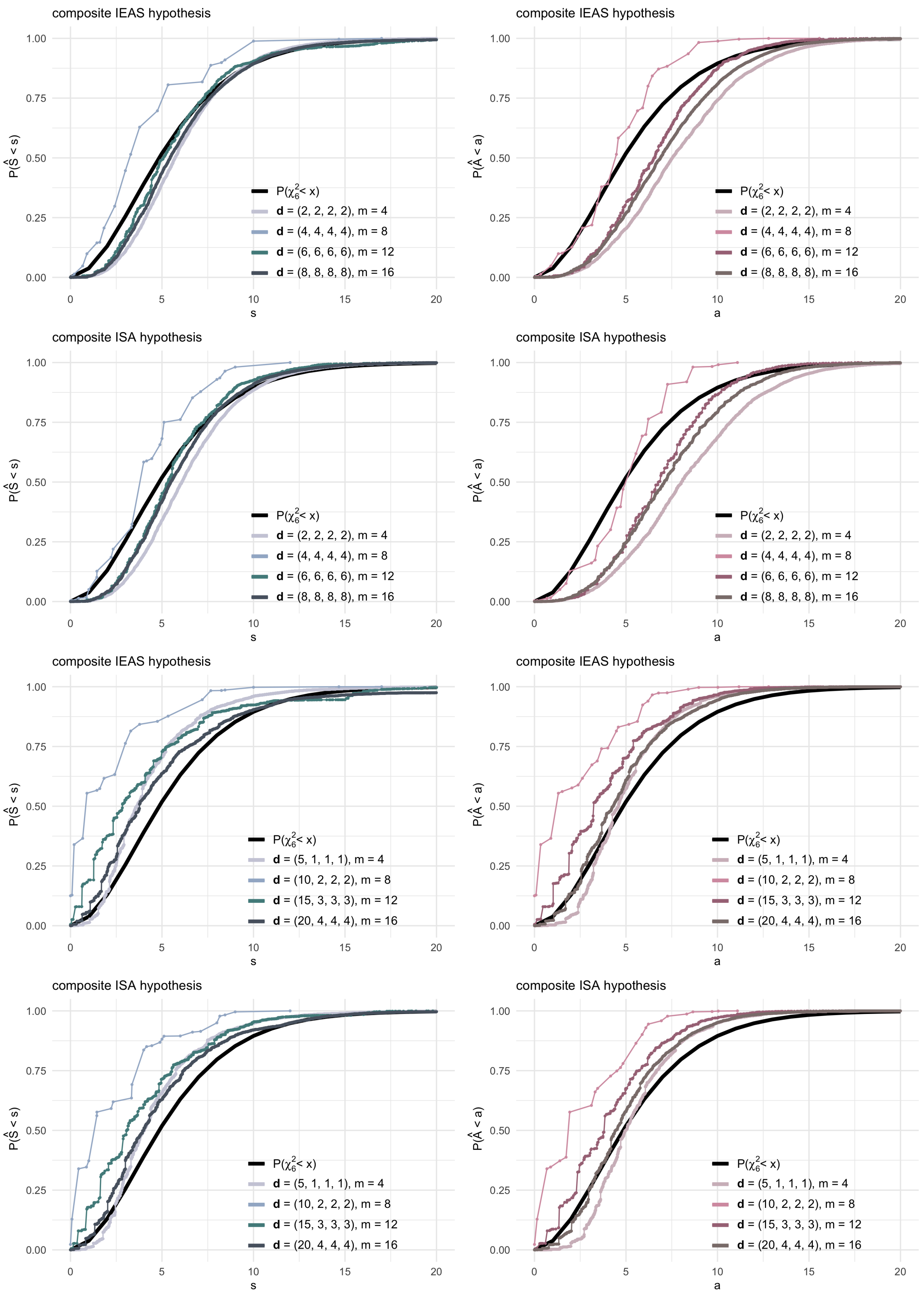

Figure 18. Null and non-null distributions of $\hat{S}$ and $\hat{A}$ for some IEAS($\mathbf{d}$) models with flat and skew $\mathbf{d}$, and composite IEAS and ISA hypotheses when m increases.

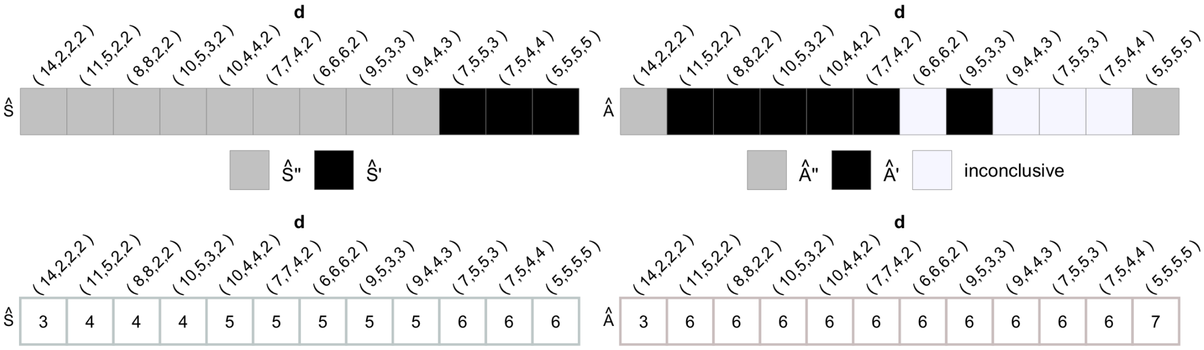

Figure 19. Preferred adjusted test statistics (top) and the degrees of freedom for preferred adjusted χ^2-distribution (bottom) for $\hat{S}$ (left) and $\hat{A}$ (right), when some composite IEAS hypotheses are tested against IEAS($\mathbf{d}$) models for multigraphs with $n = 4$ and $m = 10$.

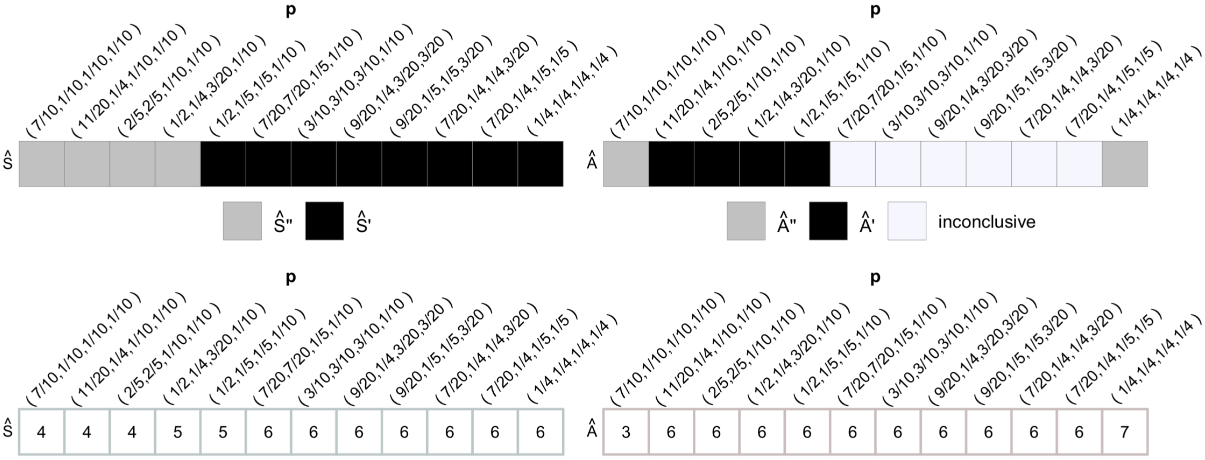

Figure 20. Preferred adjusted test statistics (top) and the degrees of freedom for preferred adjusted χ^2-distribution (bottom) for $\hat{S}$ (left) and $\hat{A}$ (right), when some composite ISA hypotheses are tested against ISA($\mathbf{p}$) models for multigraphs with $n = 4$ and $m = 10$.

4. Composite IEA hypotheses against RSM models

Figures with respect to section 5.4 in main article where composite IEA multigraph hypotheses are tested against RSM models.

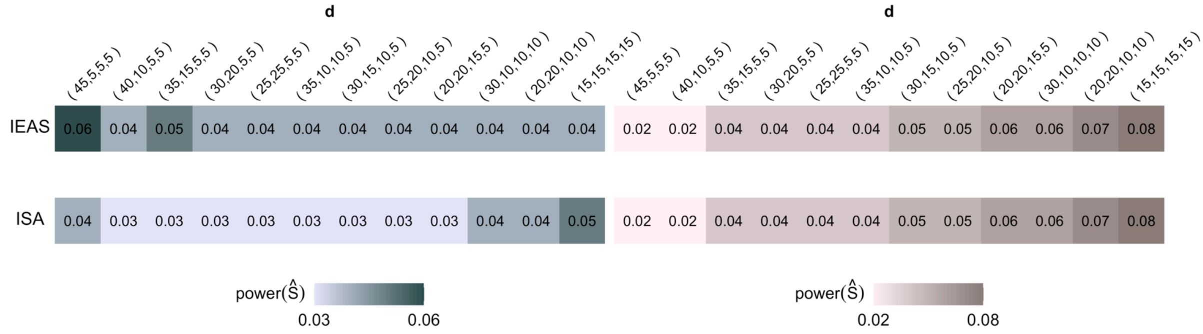

Figure 21. Power according to test statistics $\hat{S}$ and $\hat{A}$ when some composite IEAS and ISA hypotheses are tested against RSM($\mathbf{d}$) models for multigraphs with $n = 4$ and $m = 30$. The significance level for the asymptotic χ^2_6-distribution is 0.04.

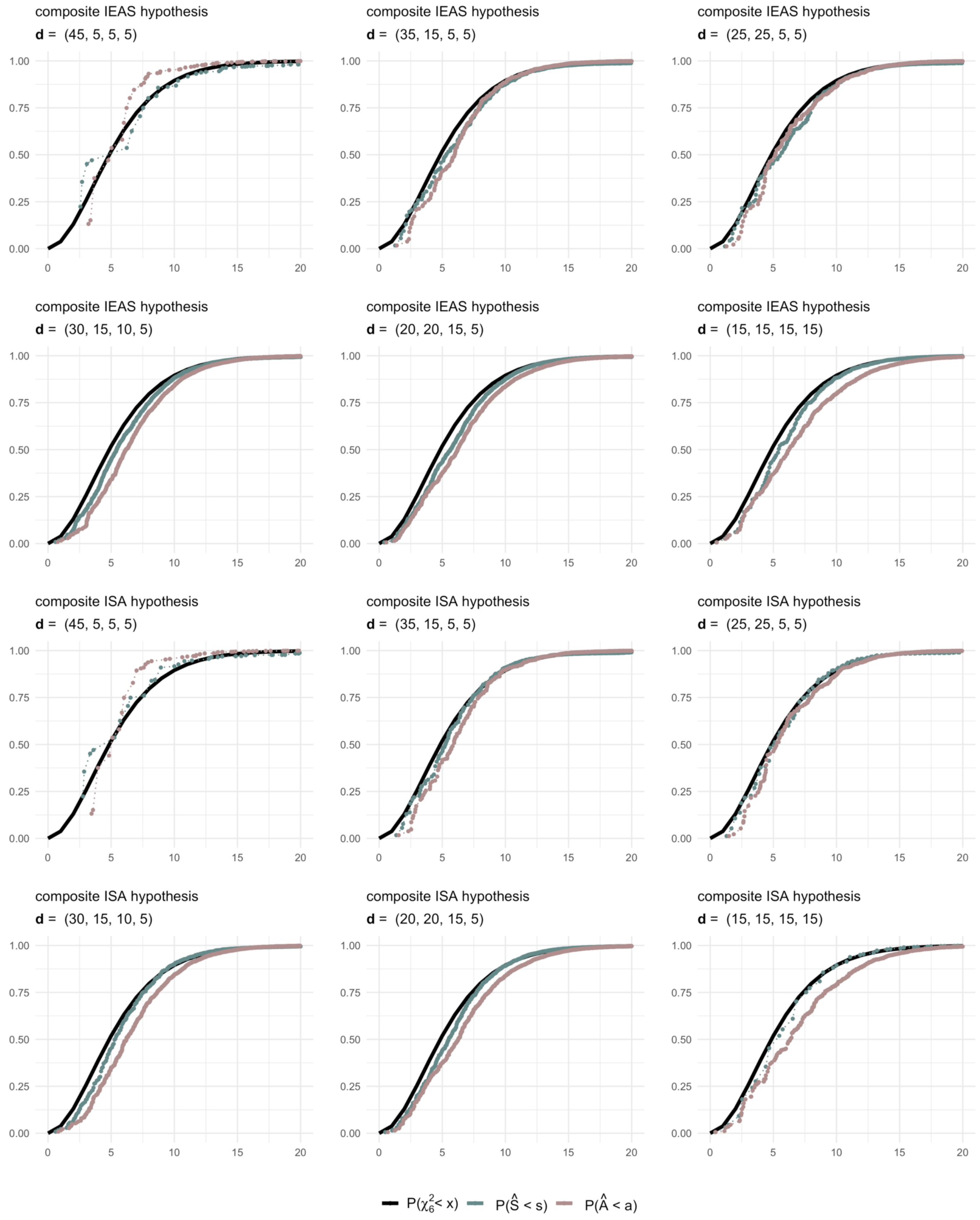

Figure 22. Non-null distributions of $\hat{S}$, $\hat{A}$, and the χ^2_6-distribution when some composite IEAS and ISA hypotheses tested against RSM($\mathbf{d}$) models for multigraphs with $n = 4$ and $m = 30$.

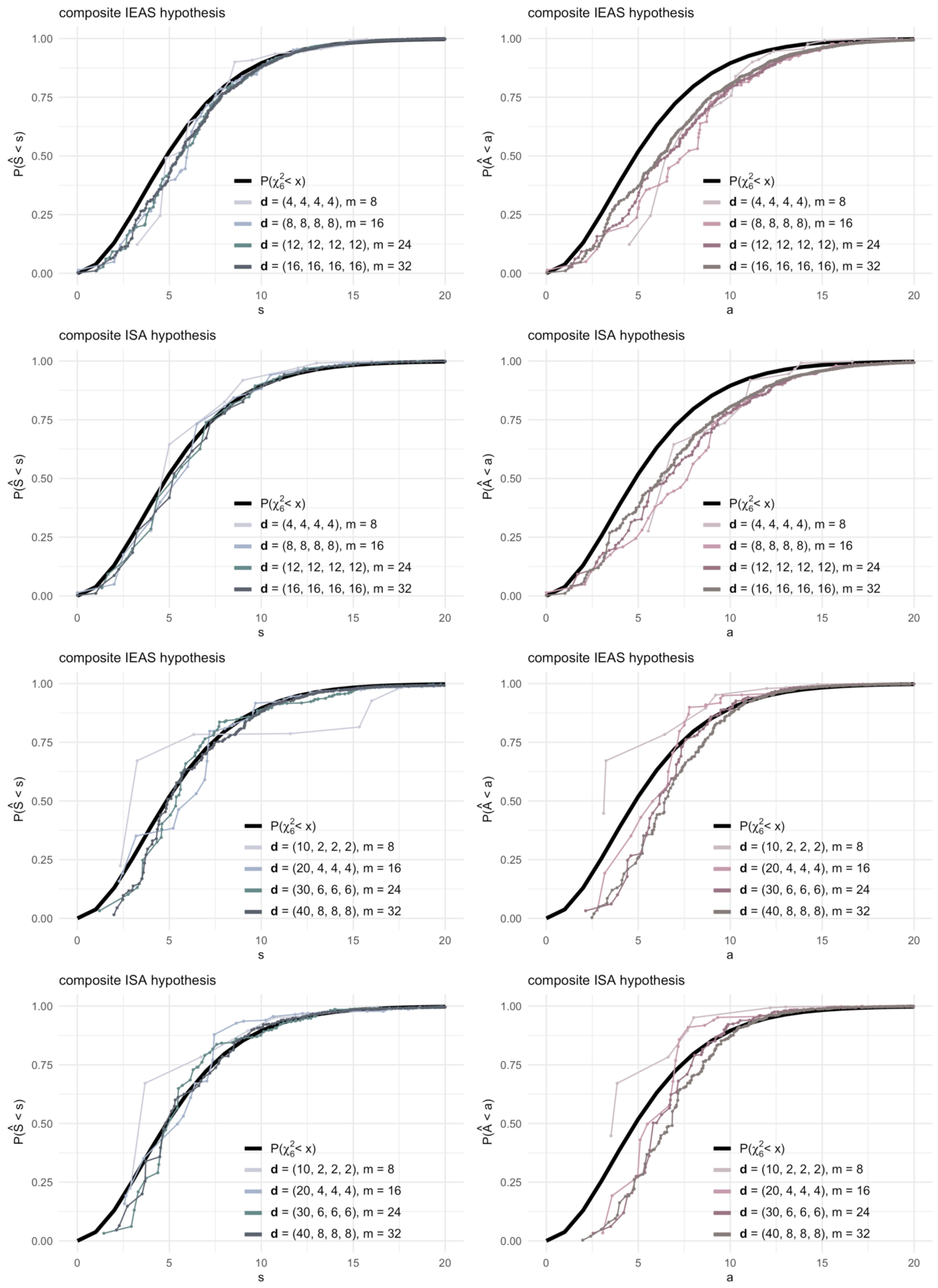

Figure 23. Non-null distributions of $\hat{S}$ and $\hat{A}$ for some RSM($\mathbf{d}$) models with flat and skew $\mathbf{d}$, and composite IEAS and ISA hypotheses when m increases.

5. Data

5.1. Florentine family networks[1].

Table 1. Dichotomized attribute variables on the 16 families representing low/high (0/1) economic W (wealth), political P (number of seats in Civic Council) and social influence S (total number of ties in larger data set).

family name	W	P	T
Acciaiuol	0	1	0
Albizzi	0	1	0
Barbadori	1	0	1
Bischeri	1	1	0
Castellan	0	1	1
Ginori	0	0	0
Guadagni	0	1	1
Lambertes	1	0	1
Medici	1	1	1
Pazzi	1	0	0
Peruzzi	1	1	1
Pucci	0	0	0
Ridolfi	0	1	0
Salviati	0	1	0
Strozzi	1	1	1
Tornabuon	1	0	0

Table 2. Edgelist showing number of financial and marital edges on multigraphs aggregated based on all three attributes, $\mathbf{m}_{PTW}$. Missing vertex pair sites in the list indicate no edges of any type occurring at those sites.

from	to	financial	marital
0 0 0	0 1 1	1	0
0 0 0	1 0 0	0	1
0 0 0	1 1 1	1	0
0 0 1	1 0 0	0	2
0 0 1	1 1 0	0	1
0 0 1	1 1 1	2	1
0 1 1	1 0 1	1	0
0 1 1	1 1 0	3	2
0 1 1	1 1 1	3	1
1 0 0	1 1 0	0	1
1 0 0	1 1 1	1	5
1 0 1	1 1 0	1	1
1 0 1	1 1 1	1	2
1 1 0	1 1 1	1	2
1 1 1	1 1 1	0	1

Table 3. Edgelist showing number of financial and marital edges on multigraphs aggregated based on pairs of attributes $\mathbf{m}_{PT}$, $\mathbf{m}_{PW}$ and $\mathbf{m}_{TW}$. Missing vertex pair sites in the list indicate no edges of any type occurring at those sites.

		$\mathbf{m}_{PT}$		$\mathbf{m}_{PW}$		$\mathbf{m}_{TW}$	
from	to	financial	marital	financial	marital	financial	marital
0 0	0 0	0	0	0	0	0	1
0 0	0 1	1	0	1	0	0	2
0 0	1 0	0	3	0	1	0	1
0 0	1 1	3	2	1	0	3	5
0 1	1 0	1	0	3	5	1	2
0 1	1 1	6	3	6	2	4	3
1 0	1 0	0	0	0	1	0	0
1 0	1 1	3	9	3	8	4	4
1 1	1 1	1	3	1	3	3	2

Table 4. Edgelist showing number of financial and marital edges on multigraphs aggregated based on single attributes $\mathbf{m}_P$, $\mathbf{m}_T$ and $\mathbf{m}_W$. Missing vertex pair sites in the list indicate no edges of any type occurring at those sites.

		$\mathbf{m}_P$		$\mathbf{m}_T$		$\mathbf{m}_W$	
from	to	financial	marital	financial	marital	financial	marital
0	0	4	12	7	6	7	5
0	1	10	8	8	11	8	13
1	1	1	0	0	3	0	2

[1]More info regarding this dataset in its original form can for example be found here. Details on the transformation and aggregation of this data set is found here

5.2. *Friendship networks in a Dutch school class*[2]

Table 5. Dichotomized actor attributes representing constant covariate 'sex' (male/female), and changing covariates 'delinquent behavior' and 'smoking' (never/at least once).

		time 1		time 2		time 3	
id	sex	delinquency	alcohol	delinquency	alcohol	delinquency	alcohol
1	0	1	0	1	1	1	1
2	0	0	1	0	1	1	1
3	0	1	1	0	0	1	1
4	1	1	0	1	0	1	0
5	1	0	0	1	0	0	0
6	0	0	0	0	0	0	0
7	0	1	1	1	1	1	1
8	0	0	0	1	0	0	0
9	0	1	1	1	1	1	1
10	0	1	1	1	1	1	1
11	0	1	1	1	1	1	1
12	0	0	0	1	1	1	1
13	1	1	1	1	1	1	1
14	1	1	0	1	1	0	1
15	0	0	0	0	0	1	0
16	1	0	0	0	0	1	0
17	0	0	0	0	0	0	1
18	1	0	0	1	0	0	0
19	0	0	0	0	0	0	0
20	0	1	1	1	1	1	0
21	0	0	0	0	0	0	0
22	1	0	0	1	0	1	1
23	0	0	0	0	0	0	0
24	1	1	0	0	0	0	0
25	1	1	0	1	1	1	1

Table 6. Edgelist showing number of reciprocated friendship ties on multigraphs aggregated based on combined attributes representing delinquent behavior, smoking and sex. Missing vertex pair sites in the list indicate no edges occurring at those sites.

from	to	time 1	time 2	time 3
0 0 0	0 0 0	7	7	2
0 0 0	0 0 1	2	0	1
0 0 0	0 1 0	0	1	1
0 0 0	1 0 0	1	3	2
0 0 0	1 0 1	1	1	1
0 0 0	1 1 0	6	2	2
0 0 0	1 1 1	0	0	2
0 0 1	0 0 1	1	1	0
0 0 1	0 1 1	0	0	2
0 0 1	1 0 1	7	0	2
0 0 1	1 1 1	0	1	3
0 1 0	1 1 0	2	5	0
0 1 0	1 1 1	0	1	0
0 1 1	1 0 1	0	0	1
0 1 1	1 1 1	0	0	1
1 0 0	1 1 0	1	0	3
1 0 1	1 0 1	1	4	0
1 0 1	1 1 0	0	2	0
1 0 1	1 1 1	0	4	2
1 1 0	1 0 1	0	2	0
1 1 0	1 1 0	4	2	4
1 1 0	1 1 1	0	1	2
1 1 1	1 1 1	0	1	2

[2] More info regarding this dataset in its original form can for example be found here. Note that time 1–3 represents wave 2–4 in original data set.
